# Electroluminescence of Tetradentate Pt(II) Complexes: O^N^N^O versus C^N^N^O Coordination

**DOI:** 10.1021/acs.inorgchem.3c00364

**Published:** 2023-03-30

**Authors:** Piotr Pander, Larissa Gomes Franca, Fernando B. Dias, Valery N. Kozhevnikov

**Affiliations:** † Faculty of Chemistry, Silesian University of Technology, Strzody 9, Gliwice 44-100, Poland; ‡ Centre for Organic and Nanohybrid Electronics, Silesian University of Technology, Konarskiego 22B, Gliwice 44-100, Poland; § Department of Physics, Durham University, South Road, Durham DH1 3LE, U.K.; ∥ Department of Applied Sciences, Faculty of Health and Life Sciences, Northumbria University, Newcastle upon Tyne, Tyne and Wear NE1 8ST, U.K.

## Abstract

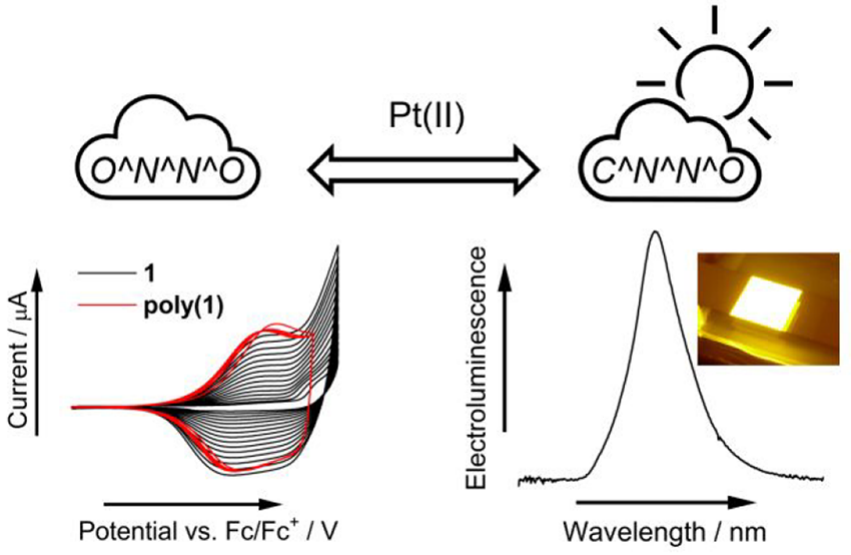

Alkylation of one of the phenolic hydroxyl groups in a salen-type tetradentate ligand changes the coordination mode from O^N^N^O to the cyclometallating C^N^N^O type. The ligand was used to synthesize a new cyclometalated luminescent Pt(II) complex **2**. While in solution the complex is poorly luminescent, in the solid state the emission is reinstated, which allowed one to evaluate complex **2** as a phosphorescent emitter in organic light-emitting diodes. **2** displays external quantum efficiency (EQE) = 9.1% and a maximum luminance of 9000 cd m^–2^ in a vacuum-deposited device. We carried out comparative analysis of photo- and electroluminescence of complex **2** with O^N^N^O complex **1** and demonstrated that the similar luminescent properties of the O^N^N^O and C^N^N^O complexes are rather coincidental because they display different excited-state landscapes. Surprisingly, the two complexes display a dramatically different electrochemical behavior, with O^N^N^O coordination leading to the formation of a stable electropolymer but C^N^N^O coordination fully preventing electropolymerization.

## Abstract 2

A rare luminescent Pt(II) complex of the C^N^N^O type displays photo- and electroluminescent properties similar to those of the related O^N^N^O-type complex. The O^N^N^O-type complex undergoes electropolymerization, giving a stable polymer film, while the complex with C^N^N^O coordination does not display similar behavior.

## Introduction

Tetradentate platinum(II) [Pt(II)] complexes are often highly luminescent, thermally stable compounds that have been successfully used as dopants in organic light-emitting devices (OLEDs).^1^ They can be used as monomolecular luminophores, where they often yield vibronically resolved luminescence, or in aggregated form as deep-red and near-infrared (NIR) emitters.^2,3^ Selected Pt(II) complexes also find applications outside of OLEDs, such as in bioimaging dyes^1^ or chemosensors.^4^ Despite being commonly recognized for their phosphorescent properties, some may display thermally activated delayed fluorescence,^5^ more known among other metal complexes, such as those of copper(I)^6^ or gold(I).^7^ This demonstrates that, despite years of research, Pt(II) complexes remain only partly understood, which poses a clear rationale for further studies in this subject.

**Scheme 1:**
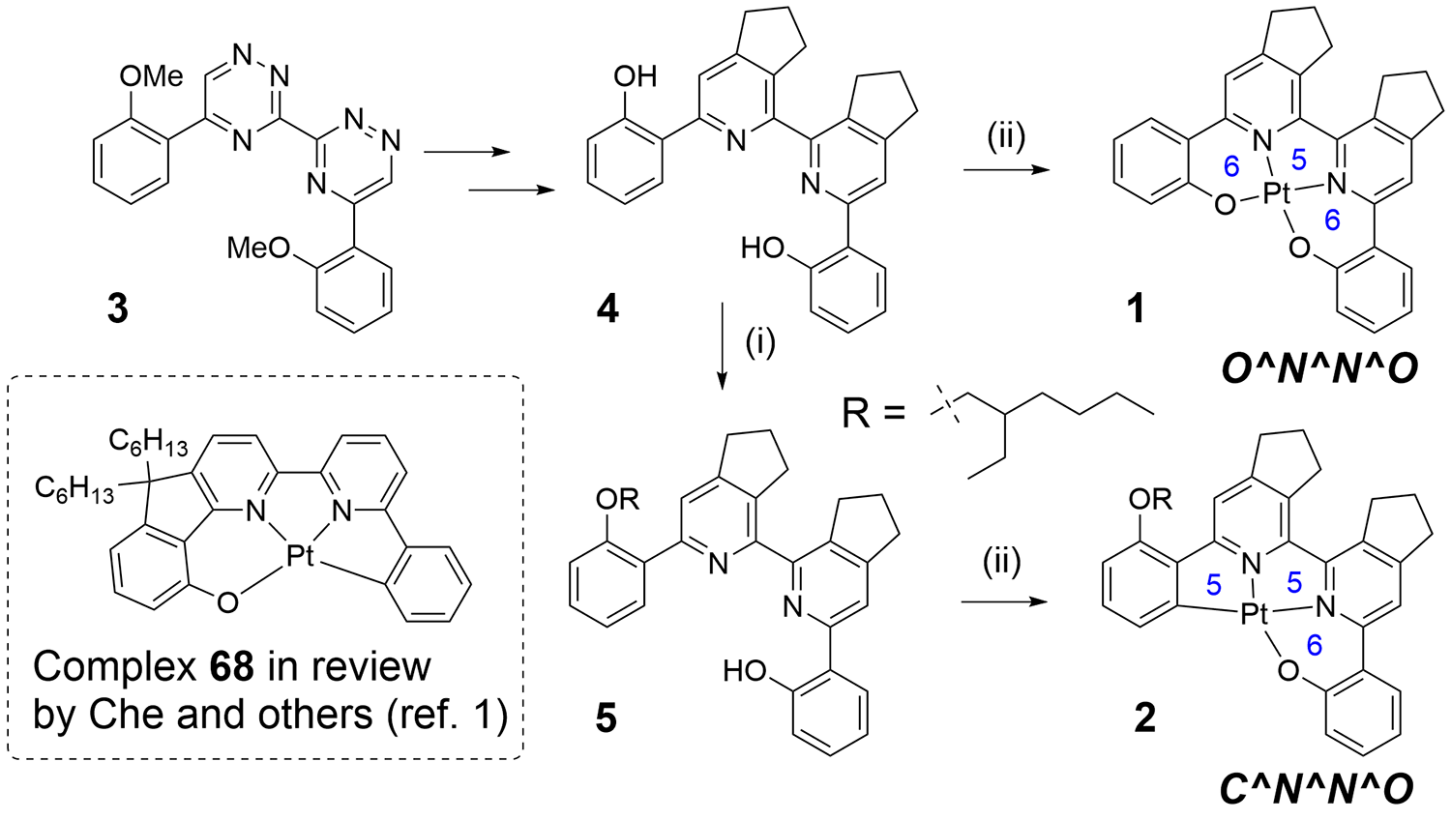
Synthesis of Pt(II) Complexes **1** and **2** (i) 1-Bromo-2-ethylhexane (1 equiv), K_2_CO_3_, DMF, 115 °C, 14 h, 37%; (ii) K_2_[PtCl_4_], AcOH, reflux, 14 h, 63% for **2**.

Pt(II) complexes stabilized by O^N^N^O-type ligands were some of the first phosphorescent materials evaluated in OLEDs.^8^ The cumulative effect of two strong σ-donor phenolate ligands deactivates the nonemissive metal-centered (MC) excited states, leading to intense luminescence. Similarly, cyclometalation is another efficient way to destabilize the MC excited states and hence to improve the luminescence. For example, tetradentate complexes of the **N^C^**N^O type with fused 6/5/5 metallocycles, in which both the phenolate and cyclometalating donor atoms are employed, are some of the best phosphorescent Pt(II) complexes known to date. The structurally similar **C^N^**N^O tetradentate Pt(II) complexes, on the other hand, are rarely investigated (the only example is complex 68 in the review by Che et al.^1^). Aggregation of square-planar Pt(II) complexes may be detrimental to their performance as OLED materials; however, certain aggregates can display intense-red and NIR luminescence.^9−12^ To minimize aggregation, bulky groups such as *tert*-butyl or twisted aromatic fragments are introduced. In our recent report, we showed that two cyclopenteno rings can be introduced through intermediary 1,2,4-triazines. In this way, the planarity of the complex is compromised, leading to improved solubility as well as a better performance in the OLED in comparison with the *tert*-butyl-substituted analogue.^13^ Here we show that a simple O-alkylation of one of the phenolates changes the coordination mode from O^N^N^O to C^N^N^O. This method, which is applicable to other salen-type ligands, improves the solubility of the complex in toluene, allowing solution fabrication of OLED devices. In this paper, we compare complexes **1** and **2** (Scheme 1) for applications in single-dopant OLEDs with emission color reminiscent of candlelight. The two complexes display surprisingly similar photoluminescent properties despite very different electronic structures. The differences are, however, more evident in their electrochemical behavior because **1** displays electropolymerization typically observed for salen-type complexes, while **2** does not.

## Results and Discussion

### Synthesis

Synthesis of the O^N^N^O-type ligands was described earlier^13^ and involved 1,2,4-triazines as intermediates (Scheme 1). The use of the 1,2,4-triazines as precursors allows an introduction of substituents by an inverse electron-demand Diels–Alder reaction. For example, two cyclopenteno units can be introduced to improve the solubility of complex **1**. To change the coordination mode from O^N^N^O to C^N^N^O, we alkylated one of the phenolic groups in **4** using 1-bromo-2-ethylhexane. The alkylation was carried out using 1 mol equiv of bromoalkene and potassium carbonate as a base in *N*,*N*-dimethylformamide (DMF) at 115 °C. We introduced the branched aliphatic chain with the aim to further improve the solubility of the complex as well as to minimize the detrimental effects of aggregation on the photophysical properties. This simple modification can be applied to other similar salen-type ligands to easily change the coordination mode from O^N^N^O with 6,5,6-metallocycles to C^N^N^O with 6,5,5-metallocycles. The alkylation, however, is not selective, and formation of the desired monoalkylated product **5** is accompanied by formation of the dialkylated product. The unreacted starting diphenol is also present in the mixture. Fortunately, the products have very different *R*_f_ values and can easily be separated by column chromatography. The isolated yield of proligand **5** was 37%. The Pt(II) complex **2** was then prepared in good yield by reaction of the proligand **5** with potassium tetrachloroplatinate in boiling acetic acid overnight.

### Photophysics

**Figure 1:**
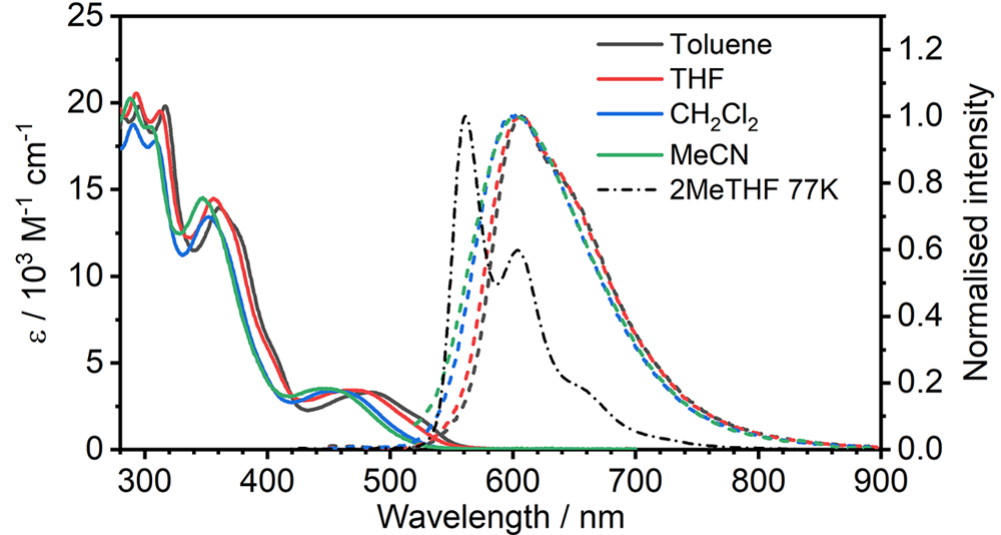
Absorption (solid lines) and PL (dashed lines; λ_ex_ = 365 nm) spectra of complex **2** recorded in solvents indicated in the figure legend at room temperature. The black dash-dotted line represents the PL spectrum recorded in 2MeTHF glass at 77 K.

To understand the differences between the C^N^N^O and O^N^N^O coordination modes, we first studied the optical properties of complex **2**. UV–vis absorption and photoluminescence (PL) spectra in solution are presented in Figure 1 and Table 1. Supplementary characteristics and PL spectra are presented in Figure S7 and Table S1. We used solvents of different polarity for the study in order to understand the excited- and ground-state properties of complex **2**. The complex displayed orange PL (λ_PL_ ≈ 600 nm) and broad spectra with insignificantly resolved vibronic progression in all solvents. We observed very clear negative solvatochromism of the absorption and PL spectra onsets with increasing solvent polarity, which is similar to behavior described earlier in analogous O^N^N^O complexes.^13^ This behavior is typical for complexes demonstrating pronounced metal-to-ligand charge-transfer character to the lowest excited state. The PL lifetime in solution is very short at room temperature, at around 0.2 μs, which is reflected also in a low Φ_PL_ ≈ 0.01–0.03, due to strong luminescence quenching. In contrast to room temperature, the PL spectrum recorded in a frozen 2-methyltetrahydrofuran (2MeTHF) glass at 77 K demonstrates a structured profile. The resultant luminescence is significantly stronger than that in liquid solutions, showing yellowish color and a longer lifetime of 11.9 μs, more typical of that presented by Pt(II) complexes.^14,15^ We observed vibronic progression of around 1400 cm^–1^, with the (0, 0) component at 561 nm being the most intense and extending out to (0, 3) at around 730 nm. The pronounced difference in the PL spectra between the liquid state and frozen glass is a manifestation of the relative flexibility of complex **2** in solution, another feature in common with the related O^N^N^O complexes.^13^

**Table 1:** Comparison of the Luminescent and Electrochemical Properties of Complexes and the OLED Performances between Analogous Complexes **1**, with O^N^N^O Coordination, and **2**, with C^N^N^O Coordination^a^

property		**1**	**2**
photophysics in toluene	λ_em_/nm	624	608
	Φ_PL_	0.24	0.02
	τ/μs	1.8	0.2
	*k*_r_/10^5^ s^–1^	1.4	0.8
	*k*_nr_/10^5^ s^–1^	0.4	49
solvent glass at 77 K	λ_em_/nm	543, 579, 638sh	561, 605, 665sh, 730sh
photophysics in the PVK/PBD matrix	λ_em_/nm	586	583
	Φ_PL_	0.49	0.30
	τ/μs	5.2	4.8
	*k*_r_/10^5^ s^–1^	0.9	0.6
	*k*_nr_/10^5^ s^–1^	1.0	1.5
electrochemistry	*E*_ox_/V vs Fc/Fc^+^	0.40	0.15
	*E*_red_/V vs Fc/Fc^+^	–1.92	–1.99
	HOMO/eV	–5.50	–5.25
	LUMO/eV	–3.18	–3.11
electroluminescence in the PVK/PBD host	λ_EL_/nm	590	582
	EQE_max_/%	10.0	7.4
	*L*_max_/cd m^–2^	7000	4000

a Data for complex **1** were reported earlier.^13^

We studied the luminescent behavior of **2** in a popular host used for solution-processed OLEDs: a blend of poly(vinylcarbazole) (PVK) and 2-(4-biphenyl)-5-(4-*tert*-butylphenyl)-1,3,4-oxadiazole (PBD) in a 60:40 (w/w) ratio. The behavior observed in a solid film is different from that in solution. PL is blue-shifted to 583 nm (Figure S7) and much more intense (Φ_PL_ = 0.30 ± 0.10). The PL decay of **2** in a film is biexponential, with an average lifetime of 4.8 μs. This dramatic change in the PL efficiency between the liquid and solid states is due to the restriction of molecular vibrations in the solid environment. We observed a very similar radiative *k*_r_ rate in toluene (8 × 10^4^ s^–1^) and the PVK/PBD film (6 × 10^4^ s^–1^), but the nonradiative decay rate *k*_nr_ is ∼30-fold larger in the former: 5 × 10^6^ s^–1^ and 1.5 × 10^5^ s^–1^, respectively. These results are also consistent with the behavior of complex **2** in 2MeTHF glass at 77 K. Complex **2** serves as an example that a low PL yield in solution does not disqualify the molecule as an efficient luminophore in the solid state, for example, for use as a luminescent dopant in OLEDs.

### Calculations

To further the understanding of the excited-state landscape of complexes **1** and **2**, we performed density functional theory (DFT) and time-dependent DFT (TD-DFT) calculations using *ORCA*(16,17) software. Ground-state (S_0_) and triplet excited-state (T_1_) geometry of the complexes was optimized using B3LYP^18,19^/def2-TZVP^20^/CPCM(toluene) for all atoms (we did not use the CPCM for the excited-state geometries), a level adequate for accurately studying metal–organic complexes. To properly model the phosphorescent properties of the complexes, we used def2-TZVP basis sets^20^ corrected by zeroth-order regular approximation^21,22^ for light atoms and a segmented all-electron relativistically contracted def2-TZVP basis set for Pt. We used the quasi-degenerate perturbation theory^23,24^ to calculate the mixing between singlet and triplet TD-DFT states (SOC-TD-DFT). This approach was previously successfully used by us to model mono- and dinuclear Pt(II) complexes.^25^ At this level of theory, we are able to model phosphorescence radiative rates^26,27^ as well as the splitting of the lowest triplet (T_1_) sublevels, in particular the splitting between sublevels 1 and 3, Δ*E*_1,3_, also known as the zero-field splitting (ZFS).^28^

#### Ground-State Geometry (S_0_)

**Figure 2:**
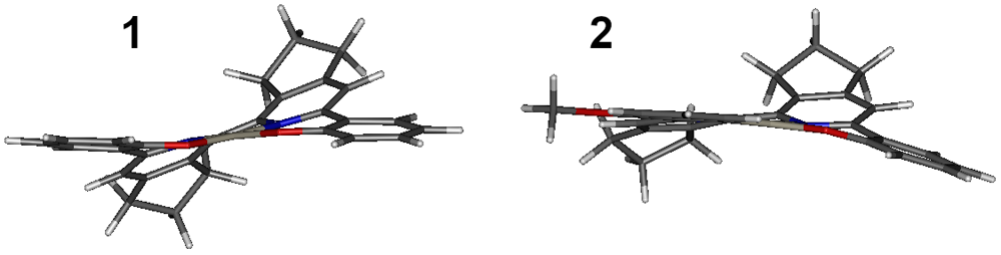
Optimized ground-state (S_0_) geometry of complexes **1** and **2** at the B3LYP/def2-TZVP/CPCM(toluene) level.

The geometry of **1** and **2** diverges from the typical planar structure of Pt complexes involving tri- or tetradentate chelating ligands (Figure 2).^8,29^ Usually, the planar configuration around the Pt(II) center is preferred, while in the case of complexes **1** and **2**, the repulsive interaction between the two cyclopentene groups forces the complex to twist. As a result, the two “outer” phenolate units are roughly planar, but the plane is twisted with respect to the “inner” 2,2′-bipyridine fragment.

**Figure 3:**
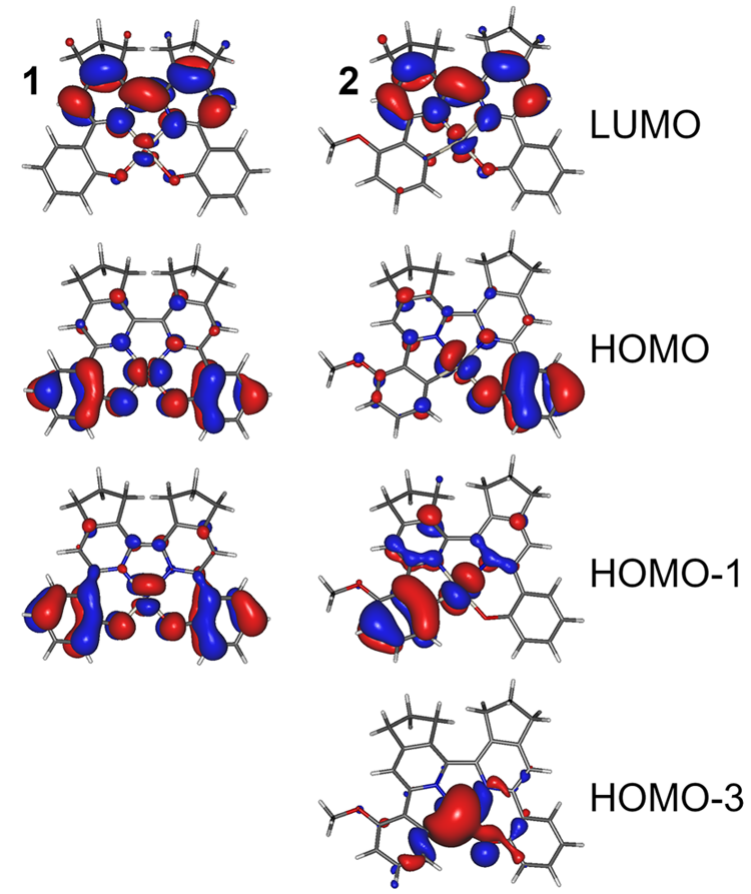
HOMO and LUMO isosurfaces at the S_0_ geometry at the B3LYP/def2-TZVP/CPCM(toluene) level for complexes **1** and **2**.

Figure 3 shows the most relevant orbitals from the standpoint of the phosphorescent properties of complexes **1** and **2**. The highest occupied molecular orbital (HOMO) and lowest unoccupied molecular orbital (LUMO) are distributed in line with the location of the electron-rich and electron-deficient fragments of the complexes. The HOMO is localized at the O-linking phenolate units and the metal center, while the LUMO is mainly on the 2,2′-bipyridine fragment. In **1**, HOMO–1 is similar to HOMO, but with a different d orbital of the central atom, while in **2**, HOMO–1 localizes on the C-linking fragment of the ligand. For both complexes, HOMO and HOMO–1 involve either a d_*xz*_ or a d_*yz*_ orbital of the Pt center, while HOMO–3 in **2** is mainly localized on the d_*z*^2^_ orbital of the metal center.

The two complexes display similar luminescent characteristics despite different types of coordination, with the predicted phosphorescence wavelengths corresponding to 666 and 649 nm, respectively for **1** and **2**. The energies of the lowest singlet states (relevant to the absorption spectrum) correspond to 620 and 597 nm, respectively.

The phosphorescent properties of the complexes originate from the mixing of singlet and triplet states by “borrowing” their radiative rates.^30^ The coupling between singlet and triplet states can be described by the spin–orbit coupling matrix element (SOCME), also presented as ⟨T_*n*_|*H*_SO_|S_*m*_⟩, while the most relevant couplings are with the states of relatively similar energy. The ⟨T_1_|*H*_SO_|S_1_⟩ in complex **1** is relatively small, at only 7 cm^–1^, consistent with the similar orbital geometry between the T_1_ (1.780 eV) and S_1_ (1.927 eV) states (both predominantly of HOMO → LUMO character). T_1_, however, couples stronger with S_2_ (2.377 eV), ⟨T_1_|*H*_SO_|S_2_⟩ = 468 cm^–1^ because the latter is predominantly of HOMO–1 → LUMO character involving a different d orbital of the metal center. Upper S_*n*_ states are at ≈1 eV or more above the T_1_, and coupling with them is less relevant. In complex **2**, T_1_ (1.908 eV) couples stronger with S_1_ (2.063 eV) than in **1**, ⟨T_1_|*H*_SO_|S_1_⟩ = 192 cm^–1^, because the two states differ in the contribution of HOMO → LUMO and HOMO–1 → LUMO transitions, while HOMO and HOMO–1 involve different d orbitals of Pt. T_1_ also couples strongly with S_2_ (HOMO–1 → LUMO, 2.514 eV), ⟨T_1_|*H*_SO_|S_2_⟩ = 839 cm^–1^, and S_3_ (HOMO–3 → LUMO, 2.588 eV), ⟨T_1_|*H*_SO_|S_2_⟩ = 1536 cm^–1^, because they both involve a different d orbital of the metal center. It appears that, although **1** and **2** display similar phosphorescent behavior, it is only coincidental because the orbital and singlet–triplet coupling patterns are drastically different.

#### Triplet Excited-State Geometry (T_1_)

Estimating the phosphorescence radiative rates from the ground-state (S_0_) geometry is usually sufficient in most cases because the cost of optimizing the T_1_ excited-state geometry is large. However, complexes **1** and **2** as relatively small systems can be subject to such optimization with a reasonable quantity of computational resources. From the calculation at the T_1_ geometry, we estimate ZFS of 8 and 16 cm^–1^ for **1** and **2**, respectively, and an identical phosphorescence radiative rate for both at 3.3 × 10^4^ s^–1^, very close to the experimental result.

The most relevant molecular orbital isosurfaces obtained from the T_1_-optimized geometries are presented in Figure S9. The T_1_–S_*n*_ coupling patterns are generally similar to those at the S_0_ geometry, but with slight changes to the ⟨T_*n*_|*H*_SO_|S_*m*_⟩ values. In **1**, we observe a relatively weak coupling between T_1_ (HOMO → LUMO, 1.366 eV) and S_1_ (HOMO → LUMO, 1.529 eV), ⟨T_1_|*H*_SO_|S_1_⟩ = 1 cm^–1^, but a significantly larger coupling constant between T_1_ and S_2_ (HOMO–1 → LUMO, 2.006 eV), ⟨T_1_|*H*_SO_|S_2_⟩ = 428 cm^–1^. Similarly, in **2**, the T_1_ (HOMO → LUMO, 1.420 eV)–S_1_ (HOMO → LUMO, 1.586 eV) SOCME is ⟨T_1_|*H*_SO_|S_1_⟩ = 152 cm^–1^, while the SOCME between T_1_ and S_2_ (HOMO–1 → LUMO, 2.219 eV) is ⟨T_1_|*H*_SO_|S_2_⟩ = 741 cm^–1^.

In conclusion, coupling between T_1_ and the upper S_*n*_ states is more significant than that between S_1_ and T_1_. The S_1_–T_1_ coupling cannot on its own explain the phosphorescent properties of Pt(II) complexes because often the two lowest states share the same orbital geometry, such as in **1**.

### Electrochemistry

**Figure 4:**
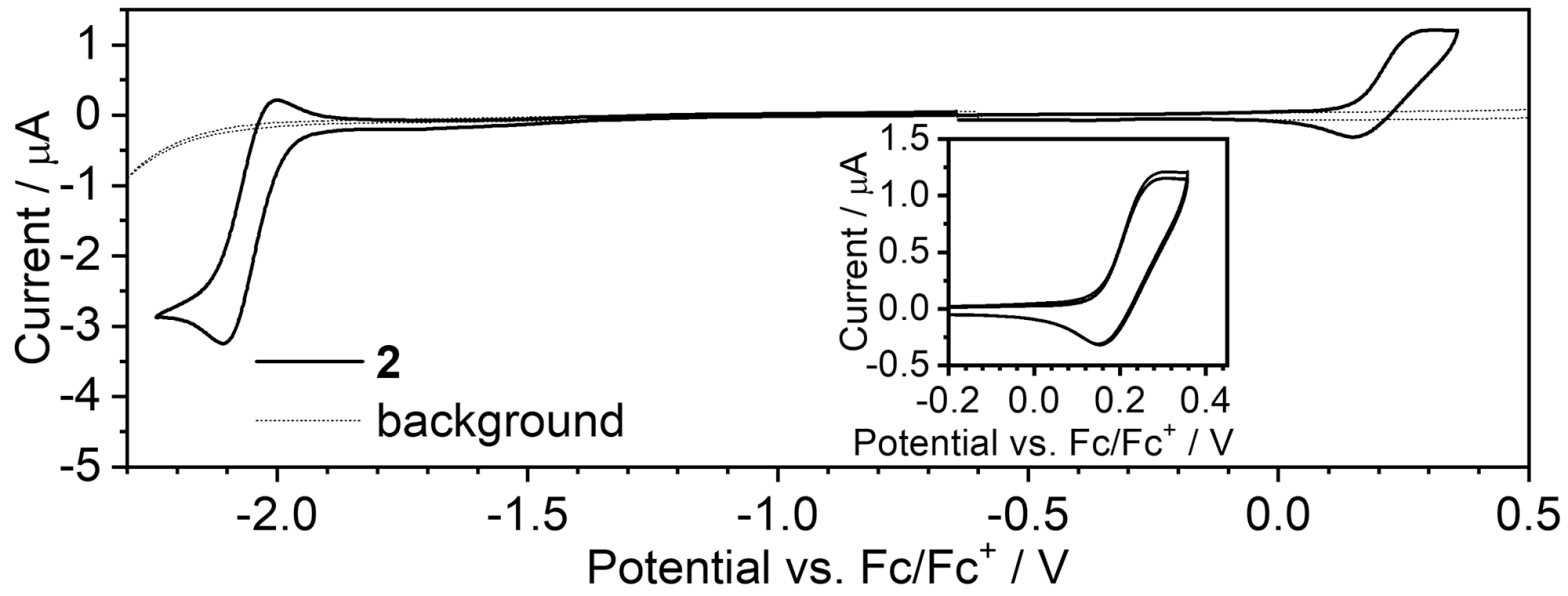
Cyclic voltammograms of **2** (*c* = 10^–3^ M) recorded in 0.1 M NBu_4_BF_4_/CH_2_Cl_2_. The inset represents three cycles recorded for the first oxidation process. Scan rate = 50 mV s^–1^.

**Figure 5:**
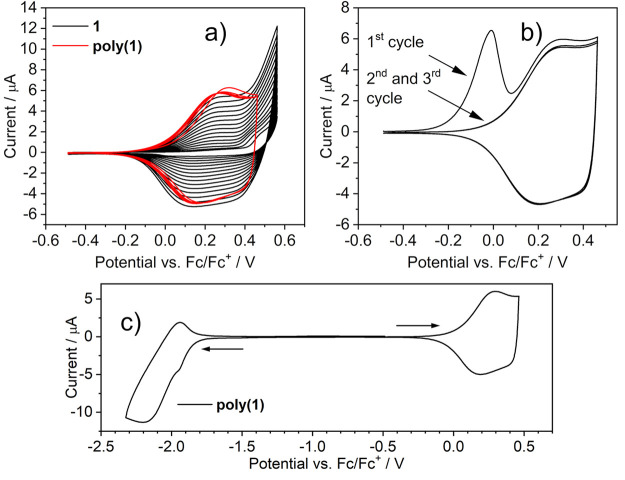
Cyclic voltammograms of **1** (*c* = 10^–3^ M) and **poly(1)** recorded in 0.1 M NBu_4_BF_4_/CH_2_Cl_2_: (a) electropolymerization and electrochemical response of the electropolymer; (b) electrochemical response of the **poly(1)** film after being subjected to a reduction cycle; (c) full redox response of the **poly(1)** film. The arrows indicate the direction of potential sweep. Scan rate = 50 mV s^–1^.

Cyclic voltammetry (Figure 4) reveals a rather atypical redox behavior of complex **2** with both reversible first oxidation and reduction processes. The reversibility of the reduction process is likely supported by stabilization of the resultant (radical) anions by the electron-deficient 2,2′-bipyridine unit of the ligand. The reduction onset potential of −1.99 V is similar to that reported earlier for the O^N^N^O analogues.^13^ Oxidation of Pt(II) complexes is typically irreversible, mainly due to involvement of the metal center in the process; thus, reversible oxidation remains rare. Oxidation at the 0.15 V onset potential in **2** most likely involves the electron-rich part of the molecule, which is lesser communicated with the metal center. In contrast to the behavior of complex **2**, complex **1** displays irreversible oxidation.^13^ We note that **2** does not display any signs of deposition of an electroactive layer on the working electrode upon oxidation. On the contrary, complex **1** appears to display deposition of a conductive layer (Figure 5a), in line with the behavior of salen-type complexes of other metals.^31,32^ This behavior of complex **1** is similar to some thiophene^33,34^ and carbazole^35,36^ derivatives, indicating that electropolymerization is taking place in this case. We deposit a thicker layer of the polymer on the electrode by repeating multiple oxidation cycles and observe an increase in the working electrode current with each cycle (Figure 5a), which is a characteristic feature of electrodeposited conductive polymers.^37^ The polymer film displays a clear and stable redox response. When subjected to reduction, it displays charge trapping (Figure 5b): negative charges become trapped within the film, only to be released upon a mildly positive potential (evident as a desorption peak at ∼0.05 V). Electropolymerization in the case of complex **1** is likely to take place through the oligomerization of cation radicals in the position para to the phenolate oxygen atom, similar to what occurs in carbazole^38^ or arylamine^39^ derivatives (para to nitrogen) and in agreement with the earlier accounts of metal-salen electropolymerization.^32^ At the same time, **2** does not undergo a process similar to that of **1**. The C-linking arm of the ligand appears to be nonreactive, so the former would be able to form dimers at most. We believe this to be the first report of a salen-type Pt(II) complex displaying electropolymerization.

### Electroluminescence

Complex **2** displays a prominent emissive behavior when used as a luminescent dopant in solution-processed and vacuum-deposited OLEDs. At first, we used a solution-processed device structure similar to that used in our earlier work^13^ to facilitate a comparison with the analogous complex **1** having O^N^N^O coordination mode. The structure of this device (Dev 1) is as follows: indium–tin oxide (ITO)|Clevios HIL 1.3 (60 nm)|PVK:PBD (60:40) co 5% **2** (70 nm)|TPBi (40 nm)|LiF (0.8 nm)|Al (100 nm). In this structure, Clevios HIL 1.3 acts as the hole injection and transport layer, while the PVK:PBD blend serves as the emissive layer. TPBi [1,3,5-tris(1-phenyl-1*H*-benzimidazol-2-yl)benzene] is the electron transport and LiF the electron injection layer. Electroluminescence of device 1 is representative for molecular PL of complex **2** indicating a perfect compatibility with the PVK:PBD host.

**Figure 6:**
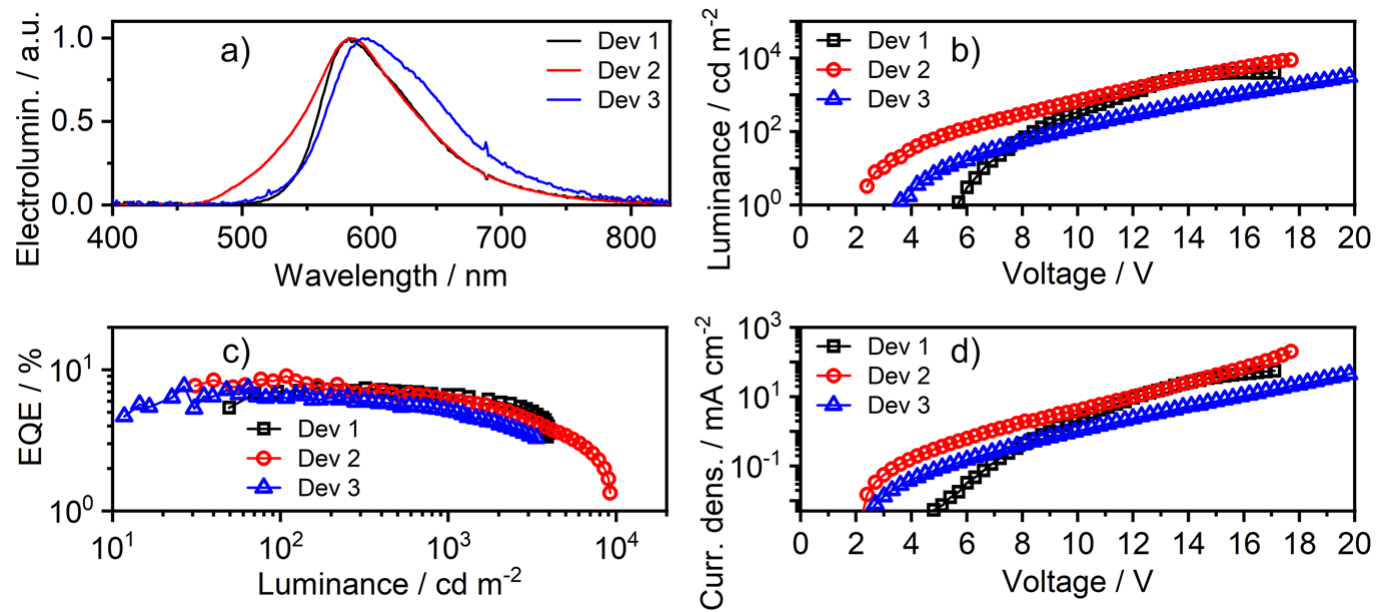
Characteristics of OLED devices 1–3: (a) electroluminescence spectra; (b) luminance versus applied voltage; (c) EQE versus luminance; (d) current density versus applied voltage.

The host used in the solution-processed OLED device 1 could not be reproduced in a vacuum-deposited structure, and a careful selection led to the use of a TCTA:PO-T2T blend^40^ in devices 2 and 3 (Figure 6 and Table 2). This led to the OLED architecture ITO|HAT-CN (10 nm)|TSBPA (40 nm)|TCTA (2 nm)|TCTA:PO-T2T (80:20) co *x* % **2** (20 nm)|PO-T2T (50 nm)|LiF (0.8 nm)|Al (100 nm), where *x* = 5 in device 2, while *x* = 10 in device 3. Here HAT-CN {dipyrazino[2,3-*f*:2′,3′-*h*]quinoxaline-2,3,6,7,10,11-hexacarbonitrile} serves as the hole injection layer, while TSBPA [4,4′-(diphenylsilanediyl)bis(*N*,*N*-diphenylaniline)] is the hole transport layer. We used the TCTA:PO-T2T blend as the host with an additional 2 nm exciton blocking layer of TCTA [4,4′,4-tris(carbazol-9-yl)triphenylamine]. PO-T2T {2,4,6-tris[3-(diphenylphosphinyl)phenyl]-1,3,5-triazine} serves as the electron transport layer and LiF as the electron injection layer. Device 2 shows a small contribution of host electroluminescence, manifested as a shoulder at around 500–550 nm, apart from the molecular luminescence from complex **2**. Increasing the concentration of **2** in the emissive layer from 5% in device 2 to 10% in device 3 leads to elimination of the host’s luminescence but leads to a red shift of the EL spectrum from 583 to 593 nm. The behavior of the EL spectrum in device 3 is likely caused by excimer or dimer formation by complex **2**, in line with some other Pt(II) complexes with a relatively planar geometry.^41^

**Table 2:** Characteristics of OLED Devices 1–3

	Dev 1	Dev 2	Dev 3
emitter	**2**	**2**	**2**
*V*_ON_/*V*^a^	6.6	3.0	5.3
*L*_max_/cd m^–2^ b	4000	9000	3400
λ_EL_/nm^c^	582	583	593
CIE 1931 (*x*, *y*)^d^	(0.55, 0.45)	(0.50, 0.48)	(0.58, 0.43)
CE_max_/cd A^–1^ e	17.1	21.4	12.9
EQE_max_/%^f^	7.4	9.1	7.6

a Turn-on voltage at 10 cd m^–2^.

b Maximum luminance.

c Electroluminescence spectrum maxima.

d Color coordinates of the electroluminescence spectrum as defined in International Commission on Illumination color space CIE 1931.

e Maximum current efficiency.

f Maximum external quantum efficiency.

The use of TCTA/PO-T2T allows minimization of the turn-on voltage *V*_ON_ from 6.6 V in the solution-processed device 1 down to 3.0 V in device 2. All three devices presented comparable characteristics, with device 2 being the most efficient, achieving an external quantum efficiency (EQE) of 9.1%. Device 2 is also the most radiant, with the maximum luminance achieving 9000 cd m^–2^. The electroluminescence color of devices 1–3 is relatively close to that of black-body radiation with temperatures between ∼1800 and 2000 K or a candle, CIE 1931 (0.52, 0.42).^42^ Complex **2** is therefore potentially useful for applications in decorative single-dopant OLED candle-imitating illumination, thanks to its relatively broadband electroluminescence.

### O^N^N^O versus C^N^N^O Coordination

In this section, we aim to gather the most important similarities and differences between O^N^N^O and C^N^N^O coordination using complexes **1** and **2** as the examples (Table 1). We first note that the two complexes are relatively similar and their behavior is alike in the solid state and OLED. The most striking difference between these two complexes is in their luminescent behavior in solution. While both complexes display comparable radiative rates in toluene, around 10^5^ s^–1^, complex **2** shows a ∼100-fold larger nonradiative decay rate, resulting in a ∼100-fold lower Φ_PL_ in **2** than in the parent complex **1**. This is a manifestation of the relative rigidity between the two luminophores because the luminescence yields in solid film are very similar. Computations suggest that the similarity in the photoluminescent behavior between **1** and **2** is only coincidental because their excited-state landscapes are rather significantly different.

Another major difference between O^N^N^O and C^N^N^O coordination can be observed with cyclic voltammetry. The C^N^N^O coordination of complex **2** does not appear to have influenced the LUMO but has clearly led to destabilization of the HOMO. Complex **2** displays a 0.25 eV higher HOMO energy than its counterpart, but the difference in the LUMO energy is only 0.07 eV. The first oxidation process in **2** is reversible, which is rare in Pt(II) complexes. A reason for this behavior of **2** might be related to stabilization of the resultant (radical) cations by the phenolate fragment of the ligand. The C^N^N^O structure of complex **2** prevents electropolymerization, which is potentially an additional factor leading to the increased stability of the initial (radical) cation in **2**. This points at only the O-linking sites of the ligand to be active in the electropolymerization process, while the C-linking sites remain inactive.

Solution-processed OLEDs produced using **1** and **2** as the emissive dopants are in general very similar to each other, with their electroluminescence spectra being alike. Complex **1** appears to slightly outcompete its C^N^N^O counterpart and displays a larger maximum luminance. C^N^N^O complex **2** still requires optimization toward reducing the nonradiative effects affecting its PL quantum yield and OLED performance. However, this simple proof-of-concept study demonstrates the feasibility of this family of luminescent complexes for use in optoelectronic devices.

## Conclusion

We have demonstrated that luminescent complexes of the C^N^N^O type can pose as prospective efficient OLED dopants, despite their modest luminescence quantum yields in solution. While the C^N^N^O proligands can easily be obtained from related O^N^N^O counterparts, this allows for additional and easy functionalization of the −OH group with alkyls or other substituents. The complex displays modest PL intensity in solution, but its luminescent performance in films is comparable with other similar complexes of the O^N^N^O type. The pseudoheteroleptic nature of complex **2** allows for better mixing between the S_*n*_ and T_1_ states, which is expressed by larger SOCMEs.

We were also able to demonstrate that the structure of complex **2** prevents electropolymerization to which **1** is susceptible. Owing to the properties of complex **1**, we were able to introduce the first salen-type Pt(II) complex to undergo electropolymerization with formation of a stable electropolymer.

Complex **2** demonstrates perfect solubility, which facilitates the fabrication of solution-processed OLEDs with a maximum EQE of 7.4% and luminances of up to 4000 cd m^–2^. Fully vacuum-deposited OLEDs yield 9.1% EQE and a maximum luminance of 9000 cd m^–2^. This performance demonstrates the feasibility of C^N^N^O complexes for OLED applications.

In conclusion, complex **2**, being one of the very few examples of C^N^N^O coordination, poses a proof-of-concept example for the usability of these types of structures as efficient luminescent materials and luminescent OLED dopants.

## Supporting Information Available

Supplementary ^1^H and ^13^C NMR spectra, experimental details, and supplementary electrochemistry, photophysics, computational, and OLED characterization results (PDF)
Cartesian coordinates (ZIP)

## Notes

A version of this manuscript has been submitted to *ChemRxiv*: 10.26434/chemrxiv-2023-sjcnw.^43^

## References

[ref1] Li K.; Ming Tong G. S.; Wan Q.; Cheng G.; Tong W.; Ang W.-H.; Kwong W.-L.; Che C. Highly Phosphorescent Platinum(II) Emitters: Photophysics, Materials and Biological Applications. Chem. Sci. 2016, 7, 1653–1673. 10.1039/C5SC03766B. 30155012 PMC6090519

[ref2] Graham K. R.; Yang Y.; Sommer J. R.; Shelton A. H.; Schanze K. S.; Xue J.; Reynolds J. R. Extended Conjugation Platinum(II) Porphyrins for Use in near-Infrared Emitting Organic Light Emitting Diodes. Chem. Mater. 2011, 23, 5305–5312. 10.1021/cm202242x.

[ref3] Cheng G.; Wan Q.; Ang W. H.; Kwong C. L.; To W. P.; Chow P. K.; Kwok C. C.; Che C. M. High-Performance Deep-Red/Near-Infrared OLEDs with Tetradentate [Pt(O ^ N ^ C ^ N)] Emitters. Adv. Opt. Mater. 2019, 7, 1801452 10.1002/adom.201801452.

[ref4] WONG K.; YAM V. Luminescence Platinum(II) Terpyridyl Complexes—From Fundamental Studies to Sensory Functions. Coord. Chem. Rev. 2007, 251, 2477–2488. 10.1016/j.ccr.2007.02.003.

[ref5] Pander P.; Daniels R.; Zaytsev A. V.; Horn A.; Sil A.; Penfold T. J.; Williams J. A. G.; Kozhevnikov V. N.; Dias F. B. Exceptionally Fast Radiative Decay of a Dinuclear Platinum Complex through Thermally Activated Delayed Fluorescence. Chem. Sci. 2021, 12, 6172–6180. 10.1039/D1SC00160D. 33996015 PMC8098751

[ref6] Romanov A. S.; Yang L.; Jones S. T. E.; Di D.; Morley O. J.; Drummond B. H.; Reponen A. P. M.; Linnolahti M.; Credgington D.; Bochmann M. Dendritic Carbene Metal Carbazole Complexes as Photoemitters for Fully Solution-Processed OLEDs. Chem. Mater. 2019, 31, 3613–3623. 10.1021/acs.chemmater.8b05112.

[ref7] Romanov A. S.; Becker C. R.; James C. E.; Di D.; Credgington D.; Linnolahti M.; Bochmann M. Copper and Gold Cyclic (Alkyl)(Amino)Carbene Complexes with Sub-Microsecond Photoemissions: Structure and Substituent Effects on Redox and Luminescent Properties. Chem. - A Eur. J. 2017, 23, 4625–4637. 10.1002/chem.201605891. 28164390

[ref8] Lin Y.; Chan S.-C.; Chan M. C. W.; Hou Y.; Zhu N.; Che C.-M.; Liu Y.; Wang Y. Structural, Photophysical, and Electrophosphorescent Properties of Platinum(II) Complexes Supported by Tetradentate N2O2 Chelates. Chem. - A Eur. J. 2003, 9, 1263–1272. 10.1002/chem.200390143. 12645015

[ref9] Wei Y.-C.; Wang S. F.; Hu Y.; Liao L.-S.; Chen D.-G.; Chang K.-H.; Wang C.-W.; Liu S.-H.; Chan W.-H.; Liao J.-L.; Hung W.-Y.; Wang T.-H.; Chen P.-T.; Hsu H.-F.; Chi Y.; Chou P.-T. Overcoming the Energy Gap Law in Near-Infrared OLEDs by Exciton-Vibration Decoupling. Nat. Photonics 2020, 14, 570–577. 10.1038/s41566-020-0653-6.

[ref10] Tuong Ly K.; Chen-Cheng R.-W.; Lin H.; Shiau Y.; Liu S.; Chou P.; Tsao C.; Huang Y.; Chi Y. Near-Infrared Organic Light-Emitting Diodes with Very High External Quantum Efficiency and Radiance. Nat. Photonics 2017, 11, 63–68. 10.1038/nphoton.2016.230.

[ref11] Pander P.; Sil A.; Salthouse R. J.; Harris C. W.; Walden M. T.; Yufit D. S.; Williams J. A. G.; Dias F. B. Excimer or Aggregate? Near Infrared Electro- and Photoluminescence from Multimolecular Excited States of N̂ĈN-Coordinated Platinum(II) Complexes. J. Mater. Chem. C 2022, 10, 15084–15095. 10.1039/D2TC01511K.

[ref12] Rossi E.; Colombo A.; Dragonetti C.; Roberto D.; Demartin F.; Cocchi M.; Brulatti P.; Fattori V.; Williams J. A. G. From Red to near Infra-Red OLEDs: The Remarkable Effect of Changing from X = -Cl to -NCS in a Cyclometallated [Pt(N∧C∧N)X] Complex (N∧C∧N = 5-Mesityl-1,3-Di-(2-Pyridyl)Benzene). Chem. Commun. 2012, 48, 3182–3184. 10.1039/c2cc16399c. 22349220

[ref13] Pander P.; Bulmer R.; Martinscroft R.; Thompson S.; Lewis F. W.; Penfold T. J.; Dias F. B.; Kozhevnikov V. N. 1,2,4-Triazines in the Synthesis of Bipyridine Bisphenolate ONNO Ligands and Their Highly Luminescent Tetradentate Pt(II) Complexes for Solution-Processable OLEDs. Inorg. Chem. 2018, 57, 3825–3832. 10.1021/acs.inorgchem.7b03175. 29537260

[ref14] Chow P. K.; Ma C.; To W. P.; Tong G. S. M.; Lai S. L.; Kui S. C. F.; Kwok W. M.; Che C. M. Strongly Phosphorescent Palladium(II) Complexes of Tetradentate Ligands with Mixed Oxygen, Carbon, and Nitrogen Donor Atoms: Photophysics, Photochemistry, and Applications. Angew. Chemie - Int. Ed. 2013, 52, 11775–11779. 10.1002/anie.201305590. 24115400

[ref15] Gildea L. F.; Williams J. A. G. Iridium and Platinum Complexes for OLEDs. Organic Light-Emitting Diodes (OLEDs); Elsevier, 2013; pp 77–113.

[ref16] Neese F. Software Update: The ORCA Program System, Version 4.0. WIREs Comput. Mol. Sci. 2018, 8 (e1327), 1 10.1002/wcms.1327.

[ref17] Neese F. The ORCA Program System. WIREs Comput. Mol. Sci. 2012, 2, 73–78. 10.1002/wcms.81.

[ref18] Becke A. D. Density-functional Thermochemistry. III. The Role of Exact Exchange. J. Chem. Phys. 1993, 98, 5648–5652. 10.1063/1.464913.

[ref19] Stephens P. J.; Devlin F. J.; Chabalowski C. F.; Frisch M. J. Ab Initio Calculation of Vibrational Absorption and Circular Dichroism Spectra Using Density Functional Force Fields. J. Phys. Chem. 1994, 98, 11623–11627. 10.1021/j100096a001.

[ref20] Weigend F.; Ahlrichs R. Balanced Basis Sets of Split Valence, Triple Zeta Valence and Quadruple Zeta Valence Quality for H to Rn: Design and Assessment of Accuracy. Phys. Chem. Chem. Phys. 2005, 7, 3297 10.1039/b508541a. 16240044

[ref21] van Lenthe E.; Baerends E. J.; Snijders J. G. Relativistic Regular Two-component Hamiltonians. J. Chem. Phys. 1993, 99, 4597–4610. 10.1063/1.466059.

[ref22] van Lenthe E.; Baerends E. J.; Snijders J. G. Relativistic Total Energy Using Regular Approximations. J. Chem. Phys. 1994, 101, 9783–9792. 10.1063/1.467943.

[ref23] Roemelt M.; Maganas D.; DeBeer S.; Neese F. A Combined DFT and Restricted Open-Shell Configuration Interaction Method Including Spin-Orbit Coupling: Application to Transition Metal L-Edge X-Ray Absorption Spectroscopy. J. Chem. Phys. 2013, 138, 204101 10.1063/1.4804607. 23742448

[ref24] de Souza B.; Farias G.; Neese F.; Izsák R. Predicting Phosphorescence Rates of Light Organic Molecules Using Time-Dependent Density Functional Theory and the Path Integral Approach to Dynamics. J. Chem. Theory Comput. 2019, 15, 1896–1904. 10.1021/acs.jctc.8b00841. 30721046 PMC6728062

[ref25] Pander P.; Zaytsev A. V.; Sil A.; Williams J. A. G.; Lanoe P.-H.; Kozhevnikov V. N.; Dias F. B. The Role of Dinuclearity in Promoting Thermally Activated Delayed Fluorescence (TADF) in Cyclometallated, N̂ĈN-Coordinated Platinum(II) Complexes. J. Mater. Chem. C 2021, 9, 10276–10287. 10.1039/D1TC02562G.

[ref26] Mori K.; Goumans T. P. M.; van Lenthe E.; Wang F. Predicting Phosphorescent Lifetimes and Zero-Field Splitting of Organometallic Complexes with Time-Dependent Density Functional Theory Including Spin-Orbit Coupling. Phys. Chem. Chem. Phys. 2014, 16, 14523–14530. 10.1039/C3CP55438D. 24664116

[ref27] Nozaki K. Theoretical Studies on Photophysical Properties and Mechanism of Phosphorescence in [Fac -Ir(2-Phenylpyridine) 3]. J. Chinese Chem. Soc. 2006, 53, 101–112. 10.1002/jccs.200600013.

[ref28] Yersin H.; Rausch A. F.; Czerwieniec R.; Hofbeck T.; Fischer T. The Triplet State of Organo-Transition Metal Compounds. Triplet Harvesting and Singlet Harvesting for Efficient OLEDs. Coordination Chemistry Review; Elsevier BV, 2011; pp 2622–2652.

[ref29] Williams J. A. G.; Beeby A.; Davies E. S.; Weinstein J. A.; Wilson C. An Alternative Route to Highly Luminescent Platinum(II) Complexes: Cyclometalation with N/\C/\N-Coordinating Dipyridylbenzene Ligands. Inorg. Chem. 2003, 42, 8609–8611. 10.1021/ic035083+. 14686833

[ref30] Baryshnikov G.; Minaev B.; Ågren H. Theory and Calculation of the Phosphorescence Phenomenon. Chem. Rev. 2017, 117, 6500–6537. 10.1021/acs.chemrev.7b00060. 28388041

[ref31] Tomczyk D.; Bukowski W.; Bester K.; Urbaniak P.; Seliger P.; Andrijewski G.; Skrzypek S. The Mechanism of Electropolymerization of Nickel(II) Salen Type Complexes. New J. Chem. 2017, 41, 2112–2123. 10.1039/C6NJ03635J.

[ref32] Łȩpicka K.; Pieta P.; Francius G.; Walcarius A.; Kutner W. Structure-Reactivity Requirements with Respect to Nickel-Salen Based Polymers for Enhanced Electrochemical Stability. Electrochim. Acta 2019, 315, 75–83. 10.1016/j.electacta.2019.05.075.

[ref33] Cabaj J.; Idzik K.; Sołoducho J.; Chyla A. Development in Synthesis and Electrochemical Properties of Thienyl Derivatives of Carbazole. Tetrahedron 2006, 62, 758–764. 10.1016/j.tet.2005.09.142.

[ref34] Zassowski P.; Golba S.; Skorka L.; Szafraniec-Gorol G.; Matussek M.; Zych D.; Danikiewicz W.; Krompiec S.; Lapkowski M.; Slodek A.; Domagala W. Spectroelectrochemistry of Alternating Ambipolar Copolymers of 4,4′- and 2,2′-Bipyridine Isomers and Quaterthiophene. Electrochim. Acta 2017, 231, 437 10.1016/j.electacta.2017.01.076.

[ref35] Data P.; Zassowski P.; Lapkowski M.; Grazulevicius J. V.; Kukhta N. A.; Reghu R. R. Electrochromic Behaviour of Triazine Based Ambipolar Compounds. Electrochim. Acta 2016, 192, 283–295. 10.1016/j.electacta.2016.01.208.

[ref36] Brzeczek A.; Ledwon P.; Data P.; Zassowski P.; Golba S.; Walczak K.; Lapkowski M. Synthesis and Properties of 1,3,5-Tricarbazolylbenzenes with Star-Shaped Architecture. Dye. Pigment. 2015, 113, 640–648. 10.1016/j.dyepig.2014.09.033.

[ref37] Data P.; Pander P.; Lapkowski M.; Swist A.; Soloducho J.; Reghu R. R.; Grazulevicius J. V. Unusual Properties of Electropolymerized 2,7-and 3,6-Carbazole Derivatives. Electrochim. Acta 2014, 128, 430–438. 10.1016/j.electacta.2013.12.108.

[ref38] Hsiao S.-H.; Wu L.-C. Fluorescent and Electrochromic Polymers from 2,8-Di(Carbazol-9-Yl)Dibenzothiophene and Its S,S -Dioxide Derivative. Dye. Pigment. 2016, 134, 51–63. 10.1016/j.dyepig.2016.06.043.

[ref39] Data P.; Pander P.; Zassowski P.; Mimaite V.; Karon K.; Lapkowski M.; Grazulevicius J. V.; Slepski P.; Darowicki K. Electrochemically Induced Synthesis of Triphenylamine-Based Polyhydrazones. Electrochim. Acta 2017, 230, 10–21. 10.1016/j.electacta.2017.01.132.

[ref40] Shafikov M. Z.; Pander P.; Zaytsev A. V.; Daniels R.; Martinscroft R.; Dias F. B.; Williams J. A. G.; Kozhevnikov V. N. Extended Ligand Conjugation and Dinuclearity as a Route to Efficient Platinum-Based near-Infrared (NIR) Triplet Emitters and Solution-Processed NIR-OLEDs. J. Mater. Chem. C 2021, 9, 127–135. 10.1039/D0TC04881J.

[ref41] Cho Y. J.; Kim S. Y.; Son H. J.; Cho D. W.; Kang S. O. Steric Effect on Excimer Formation in Planar Pt(II) Complexes. Phys. Chem. Chem. Phys. 2017, 19, 5486–5494. 10.1039/C6CP08651A. 28165085

[ref42] Jou J.-H.; Su Y.-T.; Liu S.-H.; He Z.-K.; Sahoo S.; Yu H.-H.; Chen S.-Z.; Wang C.-W.; Lee J.-R. Wet-Process Feasible Candlelight OLED. J. Mater. Chem. C 2016, 4, 6070–6077. 10.1039/C6TC01968D.

[ref43] Pander P.; Gomes Franca L.; Dias F. B.; Kozhevnikov V. N. Electroluminescence of Tetradentate Pt(II) Complexes : O ^ N ^ N ^ O versus C ^ N ^ N ^ O Coordination. ChemRxiv, 2023, 10.26434/chemrxiv-2023-sjcnw (accessed 2023–03–07). PMC10091473 36996164

