# Supplementary material for: Electroluminescence of Tetradentate Pt(II) Complexes: O^N^N^O versus C^N^N^O Coordination
Source: Inorg Chem. 2023 Mar 30;62(14):5772–9. doi: 10.1021/acs.inorgchem.3c00364 (PMC10091473; doi:10.1021/acs.inorgchem.3c00364)
Supplement: Supplementary file 1 — ic3c00364_si_001.pdf [file ic3c00364_si_001.pdf]

Supporting information

Electroluminescence of tetradentate Pt(II) complexes: O^N^N^O versus C^N^N^O coordination

Piotr Pander,[a],[b]* Larissa Gomes Franca,[c] Fernando B. Dias[c]*
and Valery N. Kozhevnikov[d]*

[a] *Faculty of Chemistry, Silesian University of Technology, Strzody 9, 44-100 Gliwice, Poland.*
E-mail: piotr.pander@polsl.pl

[b] *Centre for Organic and Nanohybrid Electronics, Silesian University of Technology, Konarskiego 22B, 44-100 Gliwice, Poland*

[c] *Department of Physics, Durham University, Durham, South Road, DH1 3LE, UK.*
E-mail: f.m.b.dias@durham.ac.uk

[d] *Department of Applied Sciences, Faculty of Health and Life Sciences, Northumbria University, Newcastle Upon Tyne, Tyne and Wear NE1 8ST, UK.*
E-mail: valery.kozhevnikov@northumbria.ac.uk

Table of contents

1. General 2
2. Synthesis 5
3. NMR spectra 6
4. Photophysics 12
5. Calculations 14
6. Electrochemistry 15
7. OLED devices 16
8. References 17

1. General

Materials and Methods

All solvents and reagents were purchased from Sigma-Aldrich, Acros Organics or Alfa-Aesar and used without further purification unless otherwise specified. Reactions were monitored by TLC using silica gel with UV_{254} fluorescent indicator. NMR spectra were recorded on a JEOL ECS400FT Delta spectrometer (399.78 MHz for ^{1}H NMR, 100.53 MHz for ^{13}C NMR). Chemical shifts are reported in parts per million (ppm) relative to tetramethylsilane as internal standard. Coupling constants (J) are measured in hertz. Multiplets are reported as follows: b = broad, s = singlet, d = doublet, dd = double doublet, t = triplet, q = quartet, qu = quintet, m = multiplet, app d = apparent doublet, app t = apparent triplet.

Photophysics

Absorption spectra of 10^{-5} M solutions were recorded with UV-3600 double beam spectrophotometer (Shimadzu). Photoluminescence (PL) spectra of solutions and films were recorded using a QePro compact spectrometer (Ocean Optics) or FluoroLog fluorescence spectrometer (Jobin Yvon). Photoluminescence decays in solution and film were recorded using nanosecond gated luminescence and lifetime measurements (from 400 ps to 1 s) using the third harmonic of a high-energy pulsed Nd:YAG laser emitting at 355 nm (EKSPLA) or with a Horiba DeltaFlex TCSPC system using a 330 nm SpectraLED or a 405 nm DeltaDiode light source. Further details are available in reference[1]. Temperature-dependent experiments were conducted using a liquid nitrogen cryostat VNF-100 (sample in flowing vapour, Janis Research) under nitrogen atmosphere, while measurements at room temperature were recorded under vacuum in the same cryostat. Solutions were degassed using five freeze-pump-thaw cycles. Thin films for photophysics were deposited from toluene solutions. The films were fabricated through spin-coating and dried under vacuum at room temperature. Solid state photoluminescence quantum yield was obtained using an integrating sphere (Labsphere) coupled with a 365 nm LED light source and QePro (Ocean Optics) detector.

Determination of photoluminescence quantum yields in solution[2]

Photoluminescence quantum yields in solution were obtained using a gradient method in which we study relation (gradient) between total photoluminescence intensity and absorbance at the excitation wavelength (same for both standard and analyte) in a range of concentrations for both analyte and standard – see equation below. We only consider data points with a constant gradient, so that the relation between photoluminescence intensity and absorbance is linear – indication of the photoluminescence yield being independent of concentration in this region.

$$\Phi_x = \Phi_{standard}\left(\frac{grad_x}{grad_{standard}}\right)\left(\frac{\eta_x^2}{\eta_{standard}^2}\right)$$

Where: Φ_x, $\Phi_{standard}$ – photoluminescence quantum yield of analyte and standard, respectively; $grad_x$, $grad_{standard}$- gradient (slope) of the linear relation between photoluminescence intensity and solution absorbance at the excitation wavelength, for analyte and standard, respectively; η_x, $\eta_{standard}$ - refractive index of solvent used for analyte and standard, respectively.

Calculations

To assist the interpretation of the experimental results, we have performed density functional theory (DFT) and time-dependent density functional theory (TDDFT) simulations with Tamm-Dancoff approximation (TDA) using ORCA 4.2.1 quantum chemistry software[3–5]. All molecular orbital (MO) iso surfaces were visualised using Gabedit 2.5.0.[6]

Geometry optimisations were performed at the B3LYP[7,8]/def2-TZVP[9] level of theory with RIJCOSX[10,11] approximation to accelerate calculations and def2/J[12] auxiliary basis set. Atom-pairwise dispersion correction with the Becke-Johnson damping scheme (D3BJ)[13,14] was included in the calculation. Single point energy calculations were performed using ZORA-corrected variants of the def2-TZVP basis set. All geometries were verified to be true energy minima by a frequency calculation. All optimisations were performed with tight SCF and geometry convergence criteria. Excited state energy of TDDFT states was calculated using the resulting S_0 or T_1 geometry. In this case relativistically corrected triple-zeta basis sets with the zeroth-order regular approximation (ZORA)[15,16] were used: ZORA-def2-TZVP[9] with the SARC/J[17] auxiliary basis for all atoms except Pt for which a segmented all-electron relativistically contracted (SARC) SARC-ZORA-TZVP[17] basis set was used. Spin-orbit coupling (SOC) calculations were performed as implemented in the ORCA software. SOC matrix elements (SOCME) and SOC-corrected excitations (SOC-TDDFT states) were computed using the same settings as for the TDDFT states. In order to accelerate the calculations RIJCOSX[10,11] approximation was used in all cases and the RI-SOMF(1X) setting was used to accelerate SOC calculations. All computations were performed using a dense grid (Grid6, GridX6).

Electrochemistry

Cyclic voltammetry was conducted in a three-electrode, one-compartment cell. All measurements were performed using 0.1 M Bu_4NBF_4 (99%, Sigma Aldrich, dried) solution in dichloromethane (ExtraDry AcroSeal®, Acros Organics). All solutions were purged with nitrogen prior to measurement and the measurement was conducted in a nitrogen atmosphere. Electrodes used in the experiment were: working (Pt disc d = 1 mm), counter (Pt wire), and reference (Ag/AgCl calibrated against ferrocene). All cyclic voltammetry measurements were performed at room temperature with a scan rate of 50 mV s^{-1}.

The ionization potential (IP) and electron affinity (EA) are obtained from onset redox potentials; these figures correspond to HOMO and LUMO values, respectively. The ionization potential is calculated from onset oxidation potential $IP = E_{ox}^{CV} + 5.1$ and the electron affinity is calculated from onset reduction potential $EA = E_{red}^{CV} + 5.1$.[18,19,20,21] An uncertainty of ±0.02 V is assumed for the electrochemical onset potentials.

OLED devices

OLEDs were fabricated by thermal evaporation or by spin-coating / evaporation hybrid method. We used pre-cleaned indium-tin-oxide (ITO) coated glass substrates with a sheet resistance of 20 Ω cm^{-2} and ITO thickness of 100 nm. The substrates were first washed with acetone and then sonicated in acetone and isopropanol, for 15 min each time. Substrates were dried with compressed air and transferred into an ozone-plasma generator for 6 min at full power. Thermally deposited layers were obtained using Kurt J. Lesker Spectros II deposition system at 10^{-6} mbar base pressure. All organic materials and aluminium were deposited at a rate of 1 Å s^{-1}. The LiF layer was deposited at a rate of 0.1–0.2 Å s^{-1}. Characterisation of OLED devices was conducted in a 10 inch integrating sphere (Labsphere) connected to a Source Measure Unit (SMU, Keithley) and coupled with a spectrometer USB4000 (Ocean Optics). Further details are available in reference.[13] Devices of 4 × 2mm pixel size were fabricated.

Substances used for OLED fabrication have been purchased from suppliers indicated in parentheses: HAT-CN – dipyrazino[2,3-f:2',3'-h]quinoxaline-2,3,6,7,10,11-hexacarbonitrile (sublimed, LUMTEC); TSBPA – 4,4'-(diphenylsilanediyl)bis(N,N-diphenylaniline) (LUMTEC); TCTA – 4,4',4-Tris(carbazol-9-yl)triphenylamine (LUMTEC); PO-T2T – 2,4,6-Tris[3-(diphenylphosphinyl)phenyl]-1,3,5-triazine (LUMTEC); PVK – poly(9-vinylcarbazole) (MW = 90 000 Da, Acros Organics), PBD -

2-(biphenyl-4-yl)-5-(4-*tert*-butylphenyl)-1,3,4-oxadiazole (Acros Organics); TPBi - 2,2',2''-(1,3,5-benzinetriyl)-tris(1-phenyl-1-*H*-benzimidazole) (LUMTEC); LiF (99.995%, Sigma Aldrich); Al pellets (99.9995%, Lesker).

Thermal evaporation. The fully thermally deposited OLEDs comprised hole injection layer: HA-TCN, hole transport layer: TSBPA, exciton blocking layer: TCTA, electron transport layer: PO-T2T, electron injection layer: LiF and cathode: Al. Optimised emissive layer comprised a blend host: TCTA (hole transport component) and PO-T2T (electron transport component) for balanced carrier ratio and improved charge injection into the emitter.

Solution processing. Hole injection layer (Heraeus Clevios HIL 1.3) was spin-coated and annealed on a hotplate at 200 ˚C for 3 min to give a 60 nm film. Emitting layer was prepared from toluene solution of PVK:PBD (60:40 w/w) with a total concentration of host 20 mg mL^{-1}. The dopants were dissolved in the solution of blend host in order to obtain final 5% (w/w) concentration in the emitting layer. The solution containing the host and the platinum complex was applied onto a substrate with HIL 1.3 layer and spun at 2500 RPM for 60 s; the substrate was then annealed at 120 ˚C for 15 min. All solutions were filtrated directly before use with a PVDF (organic solvents) syringe filter with a 0.45 μm pore size. The electron transport (TPBi) and electron injection (LiF) layers as well as cathode (Al) were thermally evaporated.

2. Synthesis

5

A mixture of **4** (1.1 g, 2.62 mmol), 1-bromo-2-ethylhexane (505 mg, 2.62 mmol) and potassium carbonate (1084 mg, 7.85 mmol) in DMF (20 mL) was stirred at 115°C for 2 days. The DMF was evaporated. The residue was treated with DCM and filtered through a short pad of silica gel using a mixture of DCM/PE/EA (5/5/1 v/v) to elute the product. By this procedure, the dark impurity on the baseline is removed. The filtrate was evaporated to dryness. The residue was treated with acetone (20 mL) and filtered. The solid on the filter was washed with acetone and dried to give unreacted diphenol **4** (201 mg). The filtrate was again evaporated to dryness and the mixture was then purified by column chromatography using silica gel as stationary phase and a mixture of PE/EA (8/1 v/v) as mobile phase. The first product to elute with the highest Rf is the symmetrical dialkylated product. Yield: 68 mg (4%). The major product is the desired ligand **5** which was the second product to elute and was isolated as an oil. Yield: 520 mg (37%). ^{1}H NMR (400.1 MHz, $CDCl_3$, Me_4Si) δ: 0.79–0.85 (6H, m), 1.18–1.24 (4H, m), 1.30–1.42 (4H, m), 1.63–1.69 (1H, m), 2.07 (2H, qu, *J* = 7.6), 2.10 (2H, qu, *J* = 7.6), 2.93 (2H, t, *J* = 7.2), 3.01 (2H, t, *J* = 7.2), 3.11 (2H, t, *J* = 7.2), 3.27 (2H, t, *J* = 7.2), 3.85 (2H, d, *J* = 5.2), 6.85 (1H, t, *J* = 7.6), 6.94 (2H, d, *J* = 8.0), 7.00 (1H, t, *J* = 7.6), 7.22–7.29 (2H, m), 7.77 (1H, s), 7.78 (1H, s), 7.79–7.82 (2H, m); ^{13}C NMR (100.6 MHz, $CDCl_3$, Me_4Si) δ: 11.3, 14.1, 23.1, 24.1, 25.1, 25.7, 29.1, 30.8, 31.7, 32.4, 32.9, 33.3, 39.6, 70.8, 112.4, 114.6, 118.3, 118.6, 119.6, 120.8, 121.5, 126.1, 129.5, 129.7, 130.8, 131.1, 137.5, 138.3, 150.2, 151.5, 153.0, 154.5, 155.1, 156.9, 157.4, 159.7; HRMS (ESI$^+$) *m/z* = 533.3174 $[M+H]^+$; calculated for $[C_{36}H_{41}N_2O_2]^+$ 533.3163.

2

A mixture of **5** (100 mg, 0.19 mmol) and potassium tetrachloroplatinate (78 mg, 0.19 mmol) in acetic acid (40 mL) was heated under reflux for 14 hours. A clear orange solution was obtained. The mixture was concentrated to a volume of approximately 5 mL, methanol (5 mL) was added and the mixture was filtered. The orange solid on the filter was washed with methanol to give the complex **2**. Yield: 87 mg (63%). ^{1}H NMR (400.1 MHz, $CDCl_3$, Me_4Si) δ: 0.96 (3H, t, *J* = 7.2), 1.03 (3H, t, *J* = 7.2), 1.37–1.68 (8H, m), 1.89 (1H, heptet, *J* = 7.2), 2.04 (4H, m), 2.82 (2H, t, *J* = 7.2), 2.83 (2H, t, *J* = 7.2), 3.03 (2H, t, *J* = 7.2), 3.07 (2H, t, *J* = 7.2), 4.01 (2H, d, *J* = 7.2), 6.64-6.70 (2H, m), 7.25–7.32 (3H, m), 7.38 (1H, dd, *J* = 8.0, 0.4), 7.72 (1H, dd, *J* = 8.0, 0.4), 7.97 (1H, s), 8.17 (1H, s); ^{13}C NMR (100.6 MHz, $CDCl_3$, Me_4Si) δ: 11.5, 14.2, 23.2, 24.3, 26.3, 26.5, 29.3, 31.1, 33.7, 33.8, 34.6, 34.7, 39.8, 70.3,107.2, 115.0,119.4, 119.7, 122.3, 124.5, 124.8, 130.1, 130.2, 130.3, 134.9, 135,4, 137.7, 150.1, 150.8, 151.6, 152.2, 157.0, 157.6, 158.2, 163.6, 164.4; HRMS (ASAP$^+$) *m/z* = 725.2663 $[M+H]^+$; calculated for $[C_{36}H_{39}N_2O_2{}^{194}Pt]^+$ 725.2638.

3. NMR spectra

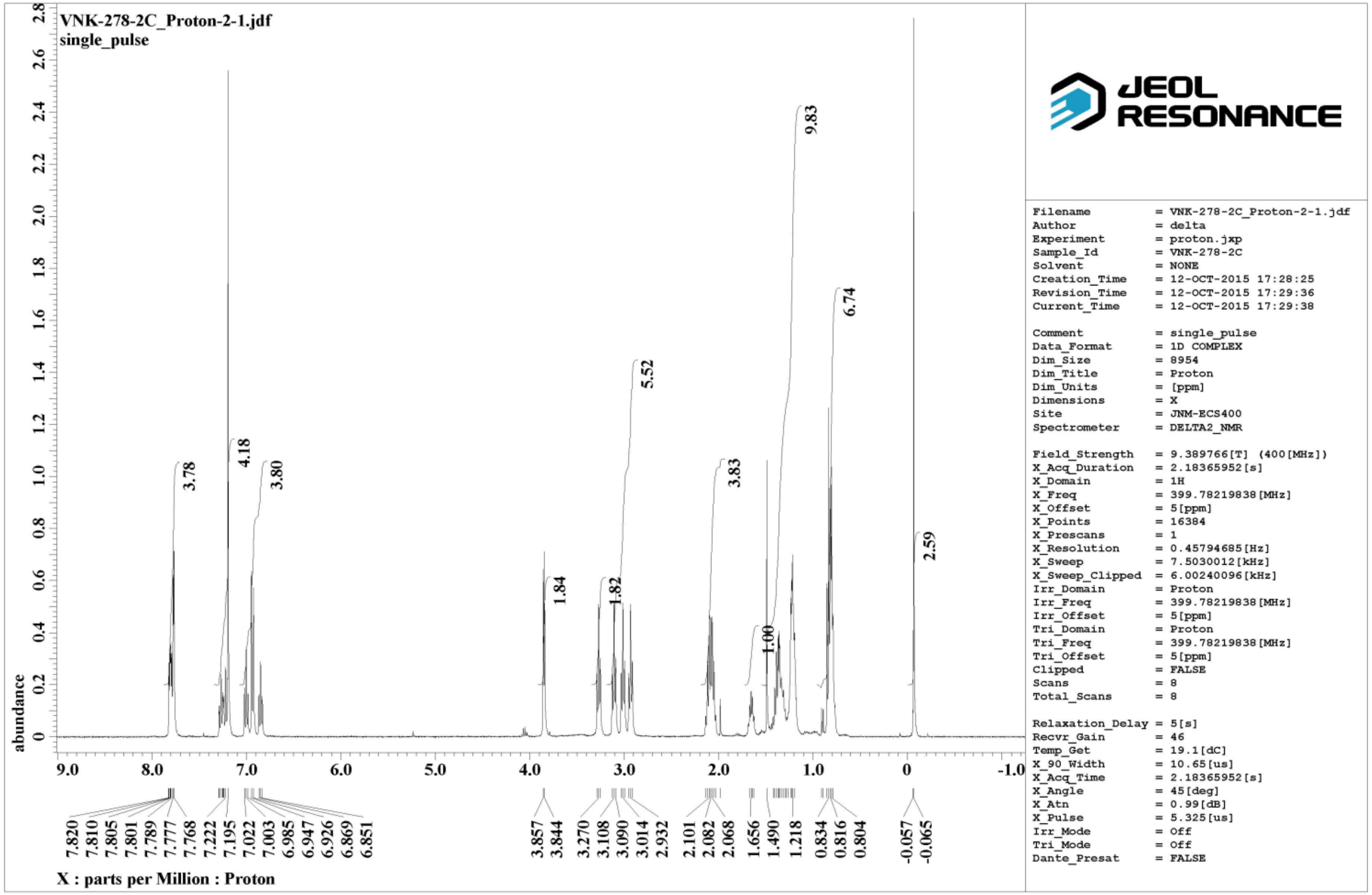

Figure S1. ^{1}H NMR spectrum of ligand **5** in $CDCl_3$.

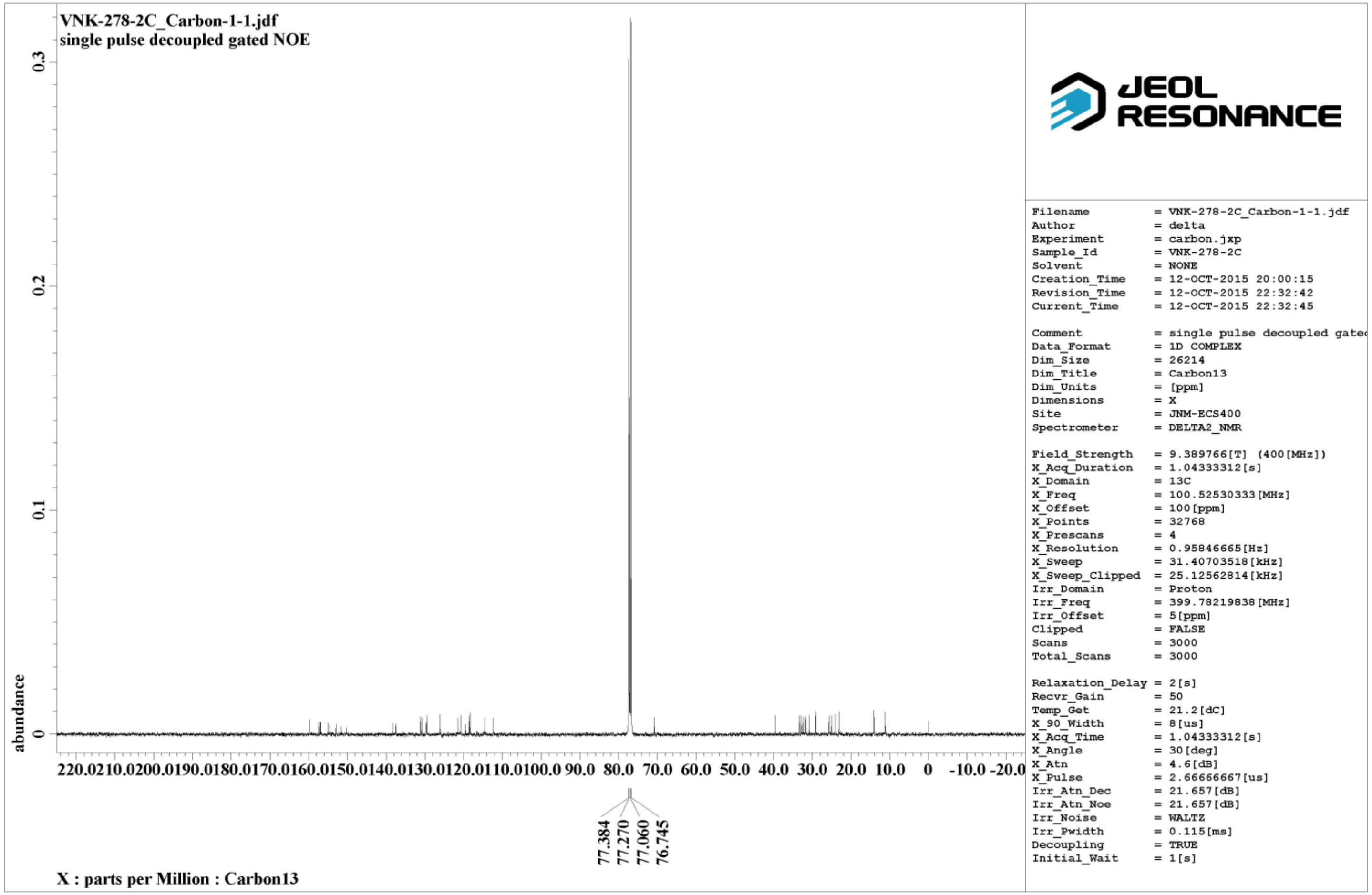

Figure S2. ^{13}C NMR spectrum of ligand **5** in $CDCl_3$.

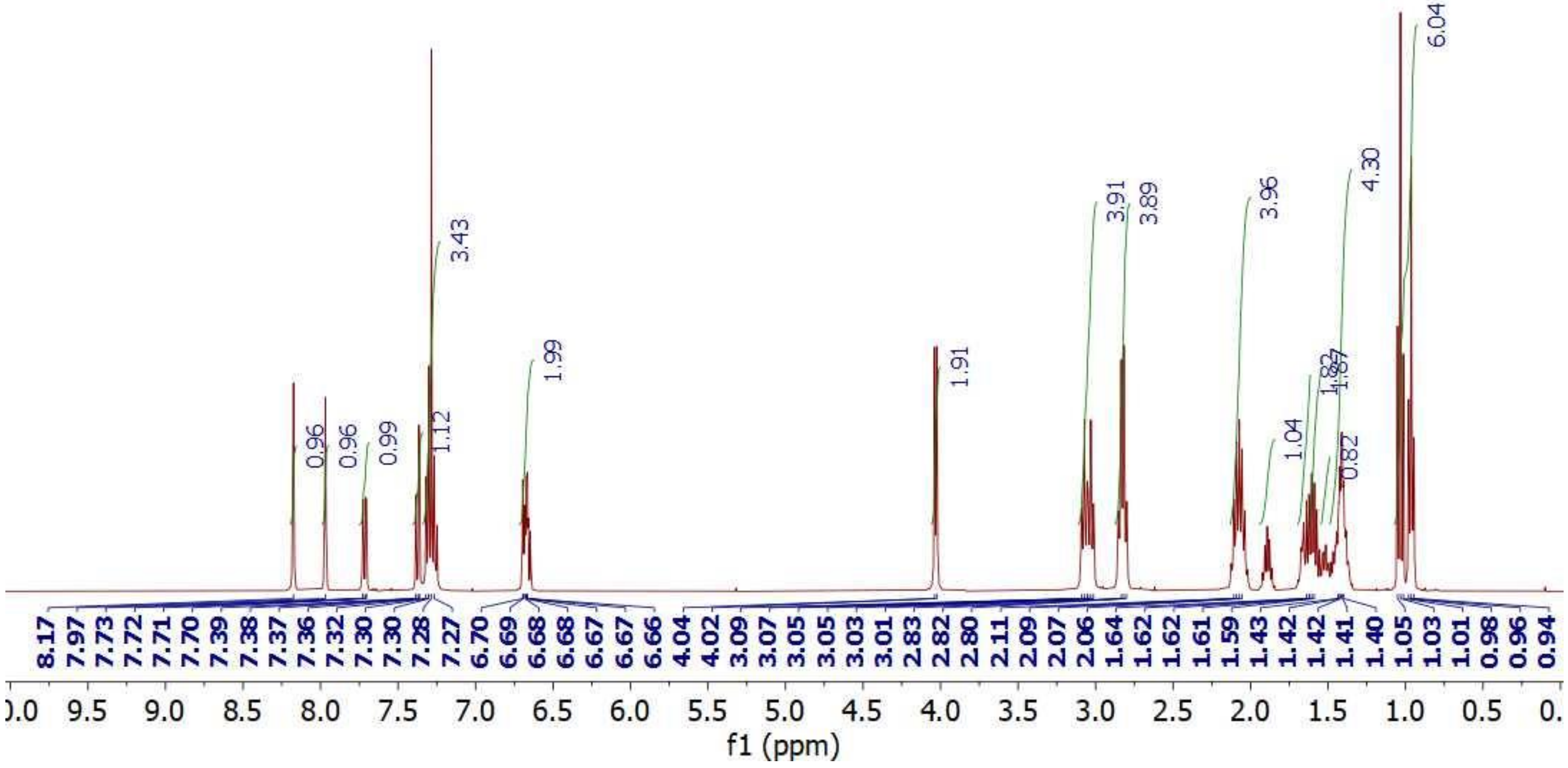

Figure S3. ^1H NMR spectrum of complex **2** in $CDCl_3$.

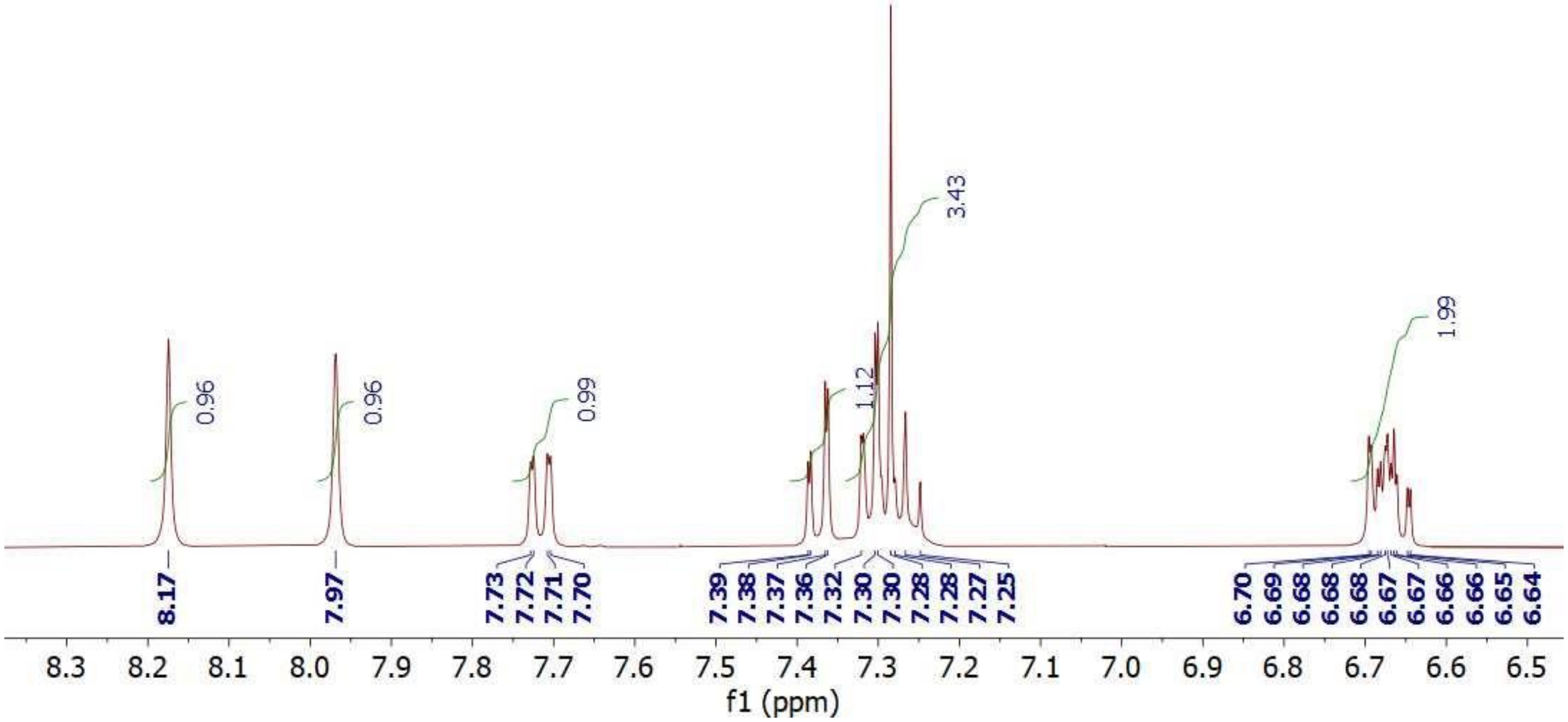

Figure S4. Expansion of the aromatic region of the ^{1}H NMR spectrum of complex **2** in $CDCl_3$.

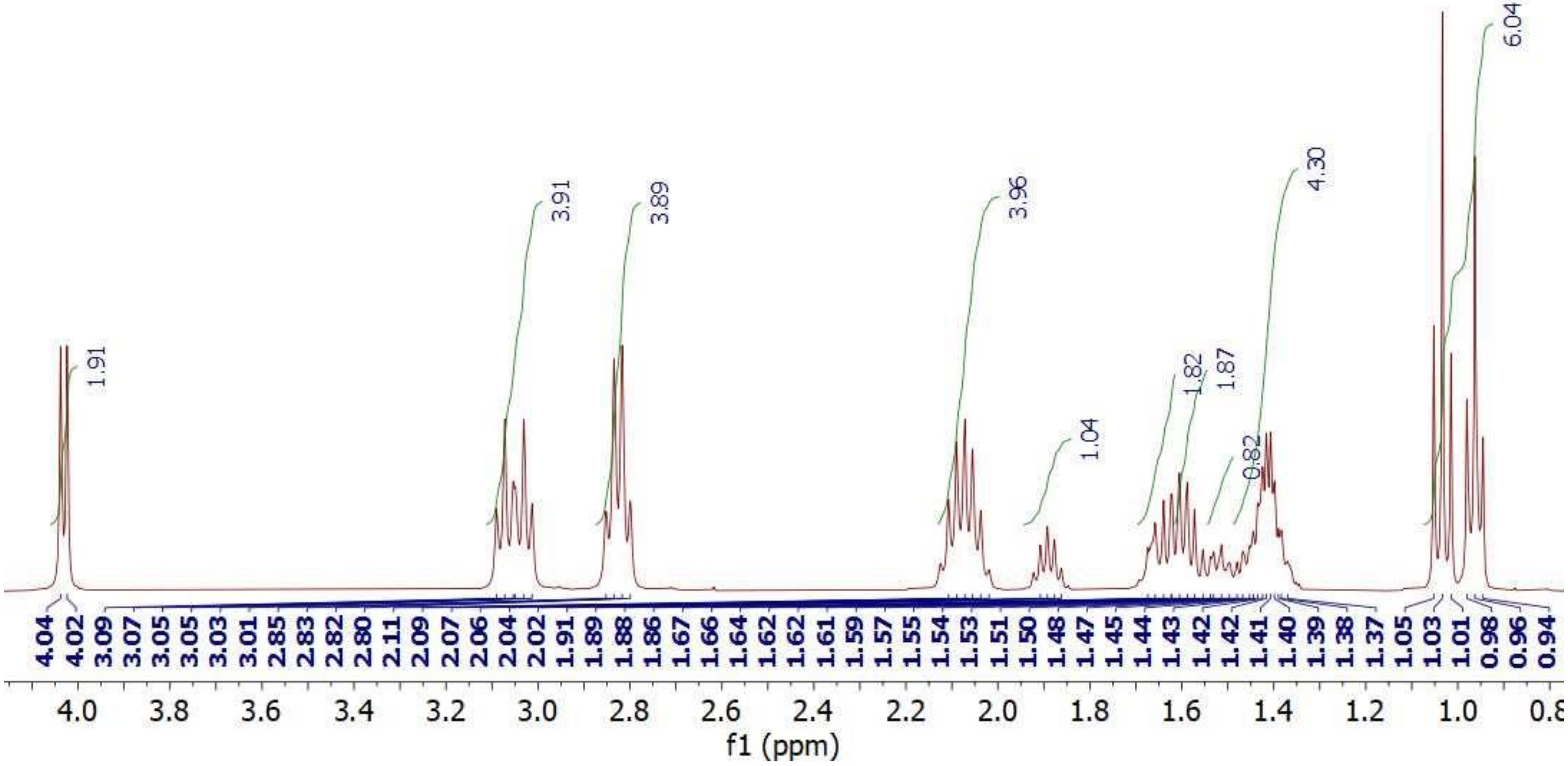

Figure S5. Expansion of the aliphatic region of the ^{1}H NMR spectrum of complex **2** in $CDCl_3$.

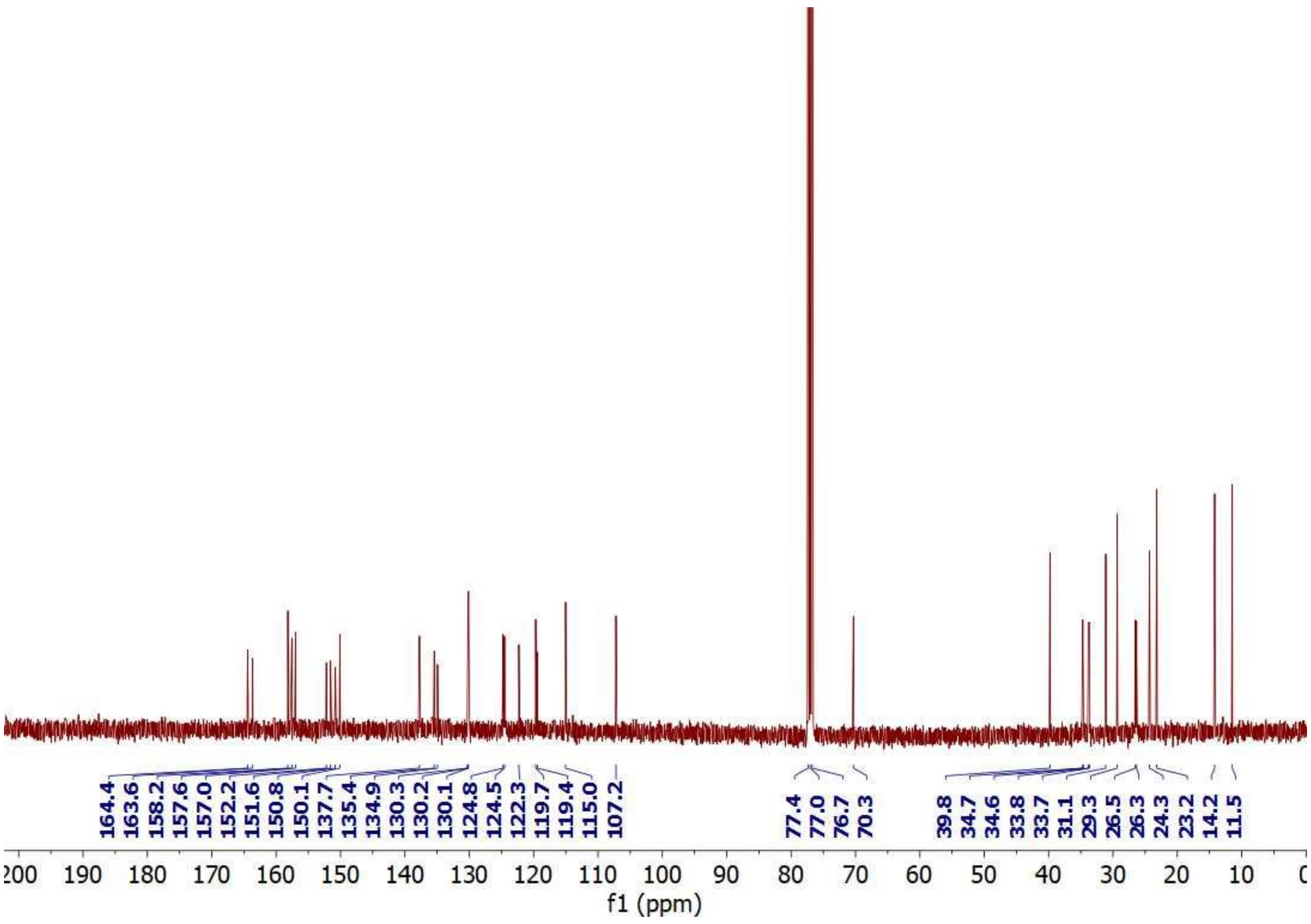

Figure S6. ^{13}C NMR spectrum of complex **2** in $CDCl_3$.

4. Photophysics

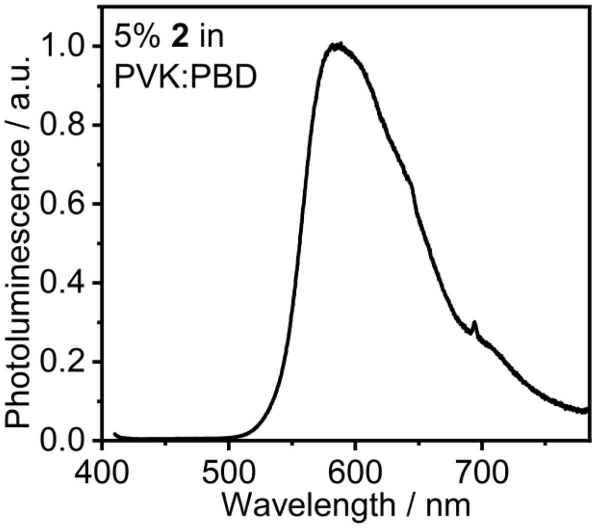

Figure S7. Photoluminescence spectrum in film of **2** (5 % w/w) doped into PVK:PBD matrix λ_{ex} = 400 nm.

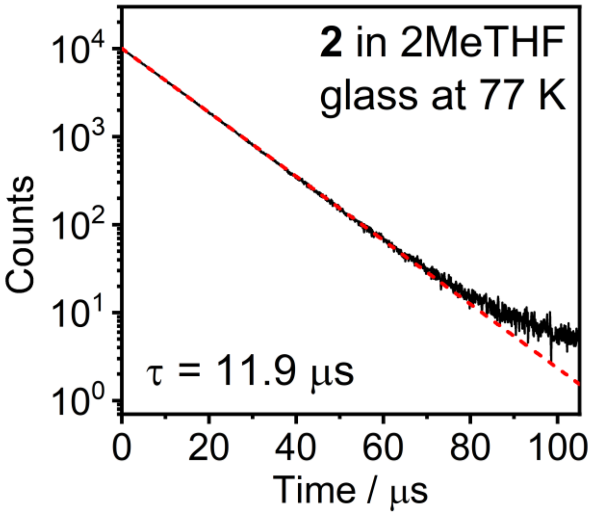

Figure S8. Photoluminescence decay trace of **2** in 2MeTHF glass at 77 K (c = 10^{-5} M), λ_{ex} = 330 nm.

Table S1. Summary of spectroscopic properties of **2** in degassed solvents at room temperature, in 2MeTHF glass at 77 K, and in solid film.

**Complex**	**Solvent / matrix**	**λ_{abs} / nm (ε / M^{-1} cm^{-1})** [a]	**λ_{em} / nm**[b]	**Φ_{PL}**[c]	**τ / µs**[d]	**k_r / 10^5 s^{-1}**[e]	**k_{nr} / 10^5 s^{-1}**[f]
**2**	Toluene	535sh (1200), 483 (3300), 410sh (4900), 378sh (12200), 360 (13900), 317 (19800), 296 (19800)	608, 645sh	0.02 ± 0.01	0.2	0.8	49
	THF	472 (3400), 403sh (5500), 374sh (12200), 356 (14500), 313 (19500), 293 (20500)	608, 640sh	0.01 ± 0.01	0.2	0.7	49
	CH_2Cl_2	457 (3400), 352 (13400), 309 (17800), 291 (18800)	603	0.01 ± 0.01	0.2	0.7	49
	Acetonitrile	449, 347, 306, 289	603	0.03 ± 0.01	0.2	1.3	49
	2MeTHF 77 K	-	561, 605, 665sh, 730sh	-	11.9	-	-
	PVK:PBD	-	583	0.30 ± 0.10	4.8	0.6	1.5

[a] Absorption maxima and molar absorption coefficients; [b] Emission maxima; [c] Photoluminescence quantum yield recorded against fluorescein in 0.1 M NaOH (Φ_{PL} = 0.90),[22] [d] Photoluminescence lifetime at room temperature; [e] Radiative rate constant, $k_r^a = \Phi_{PL}/\tau$; [f] Non-radiative rate constant, $k_{nr}^a = (1-\Phi_{PL})/\tau$.

5. Calculations

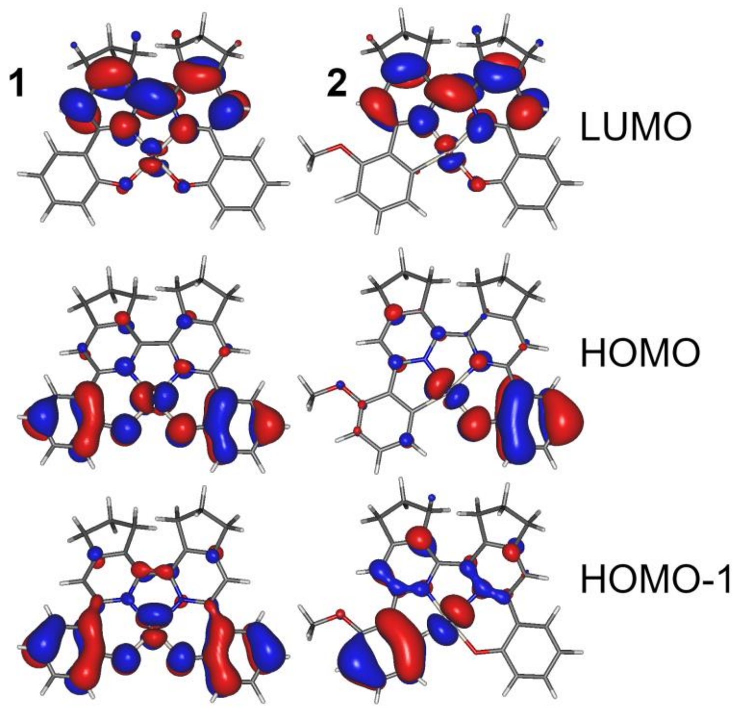

Figure S9. HOMO and LUMO iso-surfaces at T_1 geometry at the B3LYP/def2-TZVP/CPCM(toluene) level for complexes **1** and **2**.

6. Electrochemistry

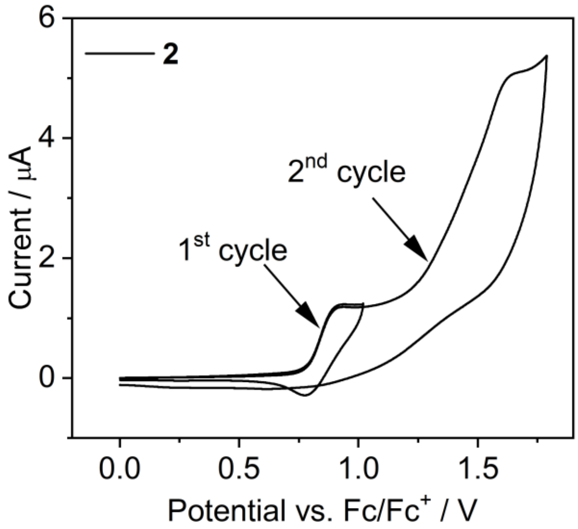

Figure S10. Cyclic voltammograms of **2** (c = 10^{-3} M) recorded at the first and second oxidation peak.

7. OLED devices

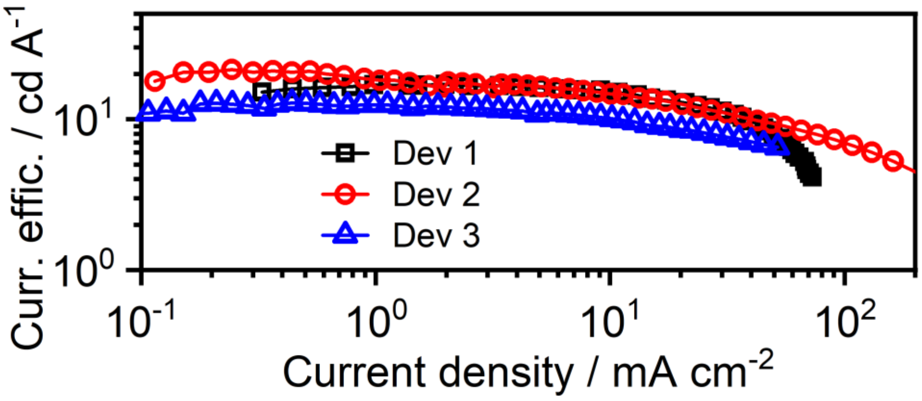

Figure S11. Current efficiency-current density characteristics of devices 1-3.

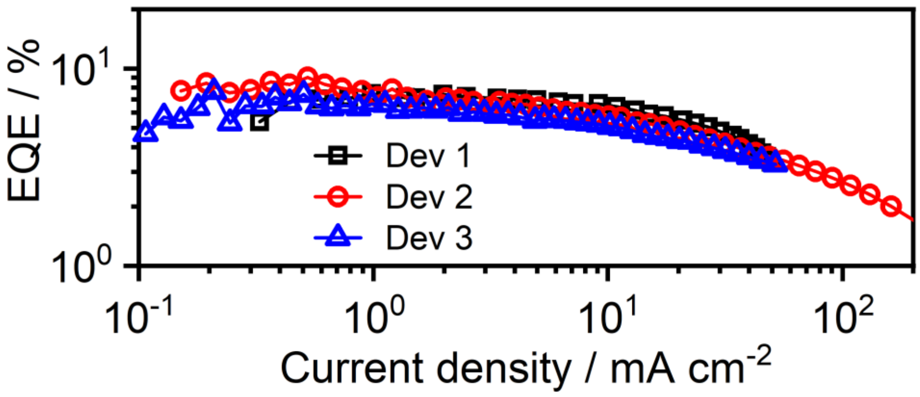

Figure S12. EQE-current density characteristics of devices 1-3.

8. References

(1) Pander, P.; Data, P.; Dias, F. B. Time-Resolved Photophysical Characterization of Triplet-Harvesting Organic Compounds at an Oxygen-Free Environment Using an ICCD Camera. *J. Vis. Exp.* **2018**, No. 142.

(2) Crosby, G. A.; Demas, J. N. Measurement of Photoluminescence Quantum Yields. Review. *J. Phys. Chem.* **1971**, *75*, 991–1024.

(3) Neese, F. Software Update: The ORCA Program System, Version 4.0. *WIREs Comput. Mol. Sci.* **2018**, *8:e1327*, 1.

(4) Neese, F. The ORCA Program System. *WIREs Comput. Mol. Sci.* **2012**, *2*, 73–78.

(5) Lehtola, S.; Steigemann, C.; Oliveira, M. J. T.; Marques, M. A. L. Recent Developments in Libxc — A Comprehensive Library of Functionals for Density Functional Theory. *SoftwareX* **2018**, *7*, 1–5.

(6) Allouche, A.-R. Gabedit-A Graphical User Interface for Computational Chemistry Softwares. *J. Comput. Chem.* **2011**, *32*, 174–182.

(7) Becke, A. D. Density-functional Thermochemistry. III. The Role of Exact Exchange. *J. Chem. Phys.* **1993**, *98*, 5648–5652.

(8) Stephens, P. J.; Devlin, F. J.; Chabalowski, C. F.; Frisch, M. J. Ab Initio Calculation of Vibrational Absorption and Circular Dichroism Spectra Using Density Functional Force Fields. *J. Phys. Chem.* **1994**, *98*, 11623–11627.

(9) Weigend, F.; Ahlrichs, R. Balanced Basis Sets of Split Valence, Triple Zeta Valence and Quadruple Zeta Valence Quality for H to Rn: Design and Assessment of Accuracy. *Phys. Chem. Chem. Phys.* **2005**, *7*, 3297.

(10) Neese, F.; Wennmohs, F.; Hansen, A.; Becker, U. Efficient, Approximate and Parallel Hartree–Fock and Hybrid DFT Calculations. A 'Chain-of-Spheres' Algorithm for the Hartree–Fock Exchange. *Chem. Phys.* **2009**, *356*, 98–109.

(11) Izsák, R.; Neese, F. An Overlap Fitted Chain of Spheres Exchange Method. *J. Chem. Phys.* **2011**, *135*, 144105.

(12) Weigend, F. Accurate Coulomb-Fitting Basis Sets for H to Rn. *Phys. Chem. Chem. Phys.* **2006**, *8*, 1057.

(13) Grimme, S.; Ehrlich, S.; Goerigk, L. Effect of the Damping Function in Dispersion Corrected Density Functional Theory. *J. Comput. Chem.* **2011**, *32*, 1456–1465.

(14) Grimme, S.; Antony, J.; Ehrlich, S.; Krieg, H. A Consistent and Accurate Ab Initio Parametrization of Density Functional Dispersion Correction (DFT-D) for the 94 Elements H-Pu. *J. Chem. Phys.* **2010**, *132*, 154104.

(15) Lenthe, E. van; Baerends, E. J.; Snijders, J. G. Relativistic Regular Two-component Hamiltonians. *J. Chem. Phys.* **1993**, *99*, 4597–4610.

(16) van Lenthe, E.; Baerends, E. J.; Snijders, J. G. Relativistic Total Energy Using Regular Approximations. *J. Chem. Phys.* **1994**, *101*, 9783–9792.

(17) Pantazis, D. A.; Chen, X. Y.; Landis, C. R.; Neese, F. All-Electron Scalar Relativistic Basis Sets for Third-Row Transition Metal Atoms. *J. Chem. Theory Comput.* **2008**, *4*, 908–919.

(18) Data, P.; Pander, P.; Lapkowski, M.; Swist, A.; Soloducho, J.; Reghu, R. R.; Grazulevicius, J. V. Unusual Properties of Electropolymerized 2,7- and 3,6- Carbazole Derivatives. *Electrochim. Acta* **2014**, *128*, 430–438.

(19) Pander, P.; Data, P.; Turczyn, R.; Lapkowski, M.; Swist, A.; Soloducho, J.; Monkman, A. P. Synthesis and Characterization of Chalcogenophene-Based Monomers with Pyridine Acceptor Unit. *Electrochim. Acta* **2016**, *210*, 773–782.

(20) Cardona, C. M.; Li, W.; Kaifer, A. E.; Stockdale, D.; Bazan, G. C. Electrochemical Considerations for Determining Absolute Frontier Orbital Energy Levels of Conjugated

Polymers for Solar Cell Applications. *Adv. Mater.* **2011**, *23*, 2367–2371.

(21) Bredas, J.-L. Mind the Gap! *Mater. Horiz.* **2014**, *1*, 17–19.

(22) Porrès, L.; Holland, A.; Pålsson, L.-O.; Monkman, A. P.; Kemp, C.; Beeby, A. Absolute Measurements of Photoluminescence Quantum Yields of Solutions Using an Integrating Sphere. *J. Fluoresc.* **2006**, *16*, 267–273.
